# Effects of Nonionizing Millimeter-Wave on Spheroid-like Irradiated Non-Small-Cell Lung Cancer (NSCLC) Cells

**DOI:** 10.3390/ijms27125621

**Published:** 2026-06-22

**Authors:** Helena Tuchinsky, Boris Litvak, Vladimir Freydin, Firas Simaan, Rawad Said, Dhaval Patel, Yosef Pinhasi, Asher Yahalom, Stella Liberman-Aronov

**Affiliations:** 1 Institute of Personal Medicine, Ariel University, Ariel 40700, P.O.B. 3, Israel; helenat@ariel.ac.il (H.T.); dhaval30103@gmail.com (D.P.); 2 Department of Electrical & Electronic Engineering, Ariel University, Ariel 40700, P.O.B. 3, Israel; borisl@ariel.ac.il (B.L.); vladimirfr@ariel.ac.il (V.F.); xrayfiras@hotmail.com (F.S.); rawad.said316@gmail.com (R.S.); yosip@ariel.ac.il (Y.P.); asya@ariel.ac.il (A.Y.); 3 Center for Astrophysics, Geophysics, and Space Sciences (AGASS), Ariel University, Ariel 40700, P.O.B. 3, Israel; 4 FEL User Center, Ariel University, Ariel 40700, P.O.B. 3, Israel

**Keywords:** millimeter waves, non-ionizing radiation, 3D spheroid, lung cancer, apoptosis, clonogenic assay, W-band

## Abstract

Non-thermal millimeter-wave (MMW) irradiation represents a promising non-invasive strategy for cancer therapy, yet its effects in physiologically relevant 3D systems remain poorly defined. Here, we evaluated the biological impact of MMW exposure in 3D non-small-cell lung cancer (NSCLC) spheroids (NCI-H1299, A549) and normal WI-38 fibroblasts under active cooling to suppress bulk heating. We demonstrate that cellular responses are governed primarily by power density (PD), irradiation geometry, and genotype-dependent susceptibility. High-PD pyramidal horn (PH) irradiation (~4.9 mW/cm^2^) induced rapid apoptosis, metabolic collapse, and near-complete loss of clonogenic survival, whereas lower-PD waveguide (WG) irradiation (~0.6 mW/cm^2^) produced depth-limited, cumulative cytotoxicity. Surviving cancer cells exhibited robust senescence-associated growth arrest, particularly in p53-deficient NCI-H1299 cells, indicating a dual apoptotic–senescent anti-proliferative response. In contrast, WI-38 fibroblasts showed minimal apoptosis and only transient stress-associated senescence, confirming selective tumor vulnerability. Mechanistic modeling suggests that MMW energy couples to glycan-rich membrane domains, generating localized electromagnetic hotspots that trigger calcium influx, mitochondrial dysfunction, and depth-dependent apoptosis. These findings establish a mechanistic basis for selective, non-thermal MMW-induced cytotoxicity in 3D NSCLC models and support further preclinical development of MMW-based therapeutic strategies.

## 1. Introduction

Despite major advances in cancer research and clinical management, lung cancer continues to pose a profound therapeutic challenge due to its aggressive biology, late-stage diagnosis, and high mortality rates. It remains the most lethal malignancy worldwide, responsible for approximately 1.8–1.9 million deaths annually and representing a substantial proportion of global cancer mortality [1,2,3]. Lung cancer is among the most frequently diagnosed cancers, with over two million new cases reported each year, and is characterized by poor overall survival, particularly in advanced stages [1,2]. Non-small-cell lung cancer (NSCLC), the predominant histological subtype, encompasses genetically and phenotypically distinct variants, including p53-deficient, KRAS-mutant, and EGFR-mutant tumors. NSCLC exhibits variable proliferation rates, apoptotic responses, and metabolic profiles. The five-year survival rate for NSCLC remains below 25% across all stages, reflecting persistent limitations of current treatment strategies [4]. Although targeted therapies and immunotherapy have improved outcomes for selected patient populations, treatment resistance and systemic toxicity remain significant clinical obstacles [5].

Despite innovations in therapeutic approaches—including biologic agents, chemotherapeutic agents, and radiation therapy—existing treatments often demonstrate toxicity while lacking selectivity for both healthy and cancerous tissues [6]. Additionally, the unavailability of precise models that faithfully simulate the human tumor microenvironment has limited the development of effective therapies [7,8]. Traditional two-dimensional (2D) cell cultures provide insights into tumor cell growth but fail to capture tumor-stroma interactions and cell–cell interactions [7]. In vivo animal models are more relevant, but they often fail to predict clinical outcomes. They are time-consuming and may involve ethical constraints. Therefore, three-dimensional (3D) in vitro models, such as tumor spheroids, have gained increasing interest to bridge the gap between 2D cell culture and animal models. Tumor spheroids replicate the architecture of the tumor microenvironment and allow for better modeling of cell interactions and response to therapies [7,8,9].

### Millimeter-Wave Studies and Anticancer Effects

Previous studies have extensively explored the effects of millimeter-wave (MMW) irradiation on cancer cells using 2D models [10,11]. As early as 2004, Radzievsky et al. [10] demonstrated that MMW therapy could inhibit melanoma growth, and ‘naloxone’ pretreatment—a competitive opioid receptor antagonist—eliminated this effect, indicating that endogenous opioids contribute to MMW-mediated cellular responses. Broadband MMW spanning 53.57–78.33 GHz inhibited the proliferation of human melanoma cells, whereas single-frequency exposures at 51.05 GHz or 65.00 GHz did not significantly alter cell growth [11]. Low-power MMW irradiation at the frequencies 42.2 GHz and 53.57 GHz did not alter the doubling time or cell cycle of RPMI 7932 melanoma cells [6,12,13]. Broadband MMW exposure induced morphological changes associated with osmotic transmembrane balance and cellular water content [14]. More recent studies demonstrated that MMW reduces A375 melanoma cell viability by activating caspase-3 and caspase-8, promoting apoptosis [13]. Frequency- and irradiation-time-dependent antiproliferative effects were confirmed in RPMI 7932 cells [11,12].

Importantly, studies from our group have further established the selective, non-thermal anticancer effects of W-band MMW irradiation. In 2D models, we demonstrated that MMW exposure induces targeted cytotoxicity in human NSCLC(NCl-H1299) while sparing non-tumorigenic epithelial cells, supporting a degree of biological selectivity [15,16]. Additional studies revealed non-thermal biological responses across different systems, including yeast models, indicating that MMW interactions extend beyond simple thermal mechanisms [17]. Furthermore, our investigations of MMW interactions with biological tissues, including characterization of loss insertion in mouse skin and in vivo studies, demonstrated minimal toxicity in healthy mice exposed to high-power, short-pulse 101 GHz radiation, supporting the safety profile of this approach [18]. Complementary findings also showed measurable non-thermal effects of MMW irradiation on human lung cancer cells, further reinforcing its therapeutic potential [15,17,18,19].

Collectively, these studies provide mechanistic evidence for the anticancer potential of MMW irradiation in 2D models, supporting its translation to more complex 3D systems. Building on these 2D findings, recent studies [20,21,22] have demonstrated that NSCLC cells exhibit genotype-dependent sensitivity to MMW irradiation, underscoring the need to investigate selective anticancer effects in physiologically relevant 3D tumor models. Compared with conventional 2D cultures, 3D spheroids more faithfully and authentically replicate tumor architecture, including cell–cell and cell–matrix interactions, nutrient and oxygen gradients, and differential energy penetration, providing a more accurate platform for evaluating therapeutic strategies such as MMW irradiation [9,14,23]. In the present study, 3D spheroids refer to droplet-based, hemispherical, spheroid-like cell aggregates formed without extracellular matrix (ECM) support.

In this study, we irradiated NSCLC cells (NCI-H1299 and A549) in a 3D hemispherical droplet model over acute (day 2), subacute (day 5), and long-term (day 10) exposure periods, alongside noncancerous lung fibroblasts (WI-38) as controls. The MMW radiation, in the W-band (75–110 GHz), was delivered using two types of antennas: a waveguide (WG) antenna, producing a localized irradiation area (0.0437 cm^2^; ~0.6 mW/cm^2^), and a pyramidal horn (PH) antenna estimated irradiation over a larger effective area (0.33 cm^2^) but with approximately tenfold lower power density (PD) (~0.08 mW/cm^2^). To increase the PH antenna’s PD, a frequency multiplier was incorporated, and the operational frequency range was narrowed to 90–96 GHz. Using this configuration without attenuation, the PD remained ~0.08 mW/cm^2^, whereas with the multiplier and optimized setup, the PD reached ~4.9 mW/cm^2^. The simulated power distribution at the sample plane was non-uniform, exhibiting a maximum near the central axis and decreasing toward the periphery. These configurations allowed for systematic evaluation of both localized (WG) and wide-area (PH) exposure setups.

This study aimed to assess the selectivity and specificity of MMW irradiation across lung cancer subtypes, identify optimal irradiation conditions, and explore underlying cellular mechanisms in a physiologically relevant 3D context. Given the distinct tissue origins and genetic mutation profiles of lung cancer cells, we hypothesized that each type and subtype may require tailored irradiation parameters to achieve optimal therapeutic efficacy.

## 2. Results

### 2.1. 3D Cell Model of the Study and Irradiation Geometry

In previous studies, we demonstrated a significant anti-cancer effect of MMW irradiation at a PD of 0.2 mW/cm^2^ in 2D human (lung) cancer and budding yeast models (*Saccharomyces cerevisiae*) [15,17]. Importantly, MMW-induced effects were significantly stronger in cancer cells than in normal cells of the same tissue origin. In the present study, we extended these findings to a physiologically relevant 3D tumor-like model, while maintaining non-thermal exposure conditions.

**Figure 1:**
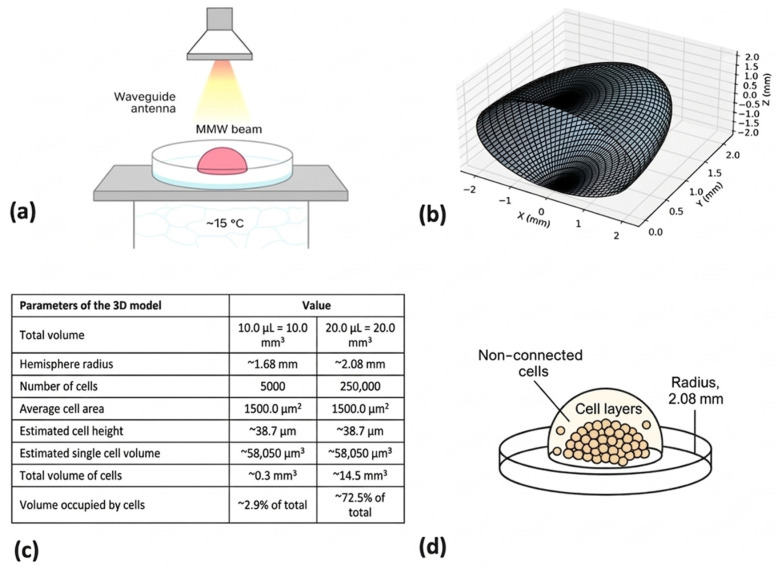
MMW irradiation setup, modeled energy distribution, and structural organization of a hemispherical tumor spheroid. (**a**) Schematic of the MMW irradiation setup. A WG antenna delivers a focused MMW beam onto a hemispherical tumor spheroid maintained at ~15 °C. (**b**) Computational 3D model showing a perfect hemisphere formed by 250,000 cells in 20 µL of medium based on geometry and total mass distribution. (**c**) Summary of the parameters for the physical (3D spheroid) model. (**d**) Structural representation of the hemispherical spheroid (radius 2.08 mm) composed of compact cell layers and peripheral non-connected cells. Panel c provides the geometric parameters of the model (e.g., volume, radius, cell density, surface curvature). Panel d depicted the structural model, which assumes a compact and uniform cell distribution, consistent with gravity-assisted self-assembly typically observed in hanging-drop or low-adhesion culture systems. The spheroid (radius: ~2.08 mm) contains multiple compact tumor cell layers forming the dome, while loosely attached, non-connected cells occupy the outer surface. These values align with the empirical 3 × 4 mm droplet footprint shown in Table 1.

**Figure 2:**
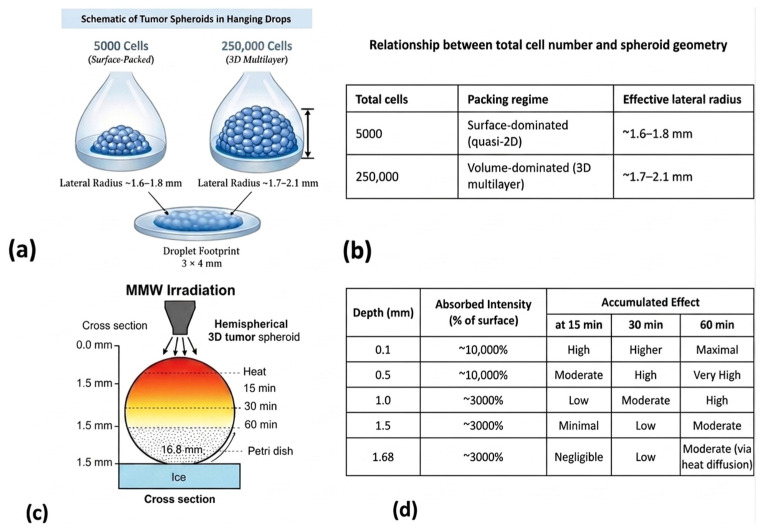
(**a**) Cell-number-dependent spheroid architecture. Schematic representation of tumor spheroids formed in hanging droplets. A small, surface-dominated (~5000 cells in 10 µL droplet) and a larger, multilayered spheroid (~250,000 cells in 20 µL) are shown. In both cases, lateral dimensions are constrained by the experimentally observed droplet footprint (~3 × 4 mm), yielding a lateral radius of approximately 1.7–1.8 mm, while vertical growth is determined by the total cell number and uniform packing. Surface-packed cells (5000 cells) vs. multilayered spheroid (250,000 cells). Vertical growth is indicated by height arrows. (**b**) Relationship between total cell number and spheroid geometry. (**c**) Distribution and attenuation of MMW irradiation in the range 75–110 GHz in a 3D tumor spheroid. (**c**) Schematic of the experimental setup showing MMW irradiation applied to a hemispherical 3D tumor spheroid (diameter: 1.68 mm) placed in a Petri dish with ice underneath to maintain a cold base. Effective heating durations vary by depth: 15 min at the surface, 30 min in the middle, and 60 min at the base. (**d**) Table summarizing depth-attenuated absorbed energy within a 1.68 mm spheroid, with maximal deposition at the surface and progressively reduced exposure toward deeper layers.

We created 3D cellular structures by depositing highly concentrated cell suspensions (10–20 µL; 5 × 10^3^–2.5 × 10^5^ cells) as hemispherical droplets onto 35 mm Petri dishes (Figure 1d and Figure 2a). Under these conditions, the cells rapidly self-assemble due to gravitational settling and spatial confinement within the droplet, forming compact, multilayered 3D aggregates. This occurs without the need for extracellular matrix (ECM) or scaffold support, with the aqueous droplet itself shaping the hemispherical geometry. These structures serve as spheroid-like 3D assemblies suitable for studying depth-dependent interactions under MMW irradiation.

We systematically evaluated the efficacy of MMW irradiation and its optimal conditions over a wide PD range (~0.6–5 mW/cm^2^) on spheroid sizes. NSCLC cell lines (NCI-H1299 and A549) were examined alongside non-cancerous (lung cell) controls (WI-38) to assess specificity. 3D multicellular models are recognized as providing a more physiologically relevant system for studying tumor biology. To characterize the geometry of the 3D system, we performed a computational spheroid modeling analysis. Figure 1 shows the MMW irradiation setup, the 3D spheroid structure model, and its properties. In Panel a, a WG antenna delivers a focused MMW irradiation beam to a hemispherical 3D tumor spheroid (maintained at approximately 15 °C). Cells with densities ranging from 5000 to 250,000 cells/10 to 20 µL in growth medium were loaded into the middle of 35 mm cell culture plates, forming a dome-shaped structure. Cooling was maintained by placing the plates on a metal table on ice, ensuring that all observed effects were non-thermal and protecting cells from thermal stress during irradiation. MMW irradiation was applied from above using a WG antenna (75–110 GHz range and PD-0.575 mW/cm^2^), enabling localized, near-field exposure of the hemispherical droplet.

Due to the physical properties of MMW, energy absorption decreases exponentially with depth according to the formula: $I=I_{0}e^{-z/l}$, where *l* represents the penetration depth (~1 mm). Consequently, superficial cell layers receive the highest energy dose, while deeper layers experience progressively attenuated exposure. Panel b presents a computational diagram that illustrates a physical model of a 3D tumor spheroid, assuming the spheroid adopts the shape of a perfect hemisphere with an average cell area of ~1500 µm^2^. The calculated radius is approximately 1.68 mm (for a 10 µL drop volume) and 2.08 mm for the two drop volumes used (20 µL).

This architecture closely mimics in vivo micro-tumor geometry and provides a controlled system for evaluating how MMW energy gradients influence apoptosis, metabolic stress, and viability across different cellular layers. 

**Table 1:** Estimated energy deposition and depth-dependent distribution in hemispherical spheroids under MMW irradiation using a WG antenna (α = 0.5).

Spheroid Radius	Duration	Total Energy Deposited (mJ)	Equivalent Power (mW)	Estimated Energy Range (Surface → Base, mJ)
1.68 mm	15 min	942–1566	1.05–1.74	942–471 → 1566–783
	30 min	1884–3132		1884–942 → 3132–1566
	60 min	3768–6264		3768–1884 → 6264–3132
2.08 mm	15 min	1494–4158	1.66–2.77	1494–747 → 4158–2079
	30 min	2988–8316		2988–1494 → 8316–4158
	60 min	5976–16,632		5976–2988 → 16,632–8316

### 2.2. Depth-Dependent Energy Deposition

Geometric parameters required for understanding cell-packing estimates for hemispherical tumor spheroids. Because the deposited droplet spreads into an elliptical rather than a circular footprint, the lateral dimensions impose a fixed geometric constraint of approximately *R*_dome_ ≈ 1.6–2.1 mm, limiting lateral expansion. As a result, additional cells accumulate vertically, forming a multilayered 3D structure. To quantify this geometry, the equivalent radius R_eq_ of the elliptical footprint was calculated as follows:

$$R_{eq}=\sqrt{ab}$$

where a = 2.0 mm and b = 1.5 mm represent the semi-axes of the ellipse. This yields:

$$R_{eq}=\sqrt{2.0\;\times 1.5}\;\approx \;1.73\;mm$$

This equivalent radius closely matches our previous calculation (1.68–1.8 mm).

Assuming a hemispherical geometry, the available curved surface area *A_hemi_* is:

$$A_{hemi}=2\pi R_{eq}^{2}$$

$$A_{hemi}=2{\left(1.73\;mm\right)}^{2}\;\approx \;18.8\;{mm}^{2}=1.88\times 10^{7}\;\mu m^{2}$$

The maximum number of cells that can occupy the hemispherical surface is given by:

$$N_{surface}=\frac{A_{hemi}}{A_{cell}}$$

Assuming an average projected cell area of ~1500 µm^2^:

$$N_{surface}=\frac{1.88\;\times 10^{4}}{1500}\;\approx \;1.25\;\times \;10^{4}\;cells$$

Thus, a droplet containing ~5000 cells per 10 µL forms a surface-packed monolayer-like spheroid, whereas a 250,000-cell spheroid in a 20 µL droplet corresponds to ~20 cell layers, forming a compact multilayered dome. Because the footprint remains constrained (~1.6–1.8 mm), increases in cell number primarily increase spheroid height rather than lateral spread.

Figure 2a schematically illustrates these differences. Although slight deviations from the idealized geometry may occur due to wetting behavior and cooling conditions, the droplet footprint remained largely stable because droplets were deposited on plastic Petri dishes cooled from below (4 °C metal support). This ensured that increases in cell number translated predominantly into vertical thickening.

Figure 2c,d illustrates the theoretical distribution of absorbed MMW energy within a hemispherical spheroid positioned on a cold base. Figure 2c shows the differential exposure of spheroid layers, with effective irradiation times of 15 min at the surface, 30 min in the middle, and 60 min at the base. Figure 2d summarizes the estimated absorbed energy within a spheroid of 1.68 mm radius as a function of irradiation duration and depth. Calculations assume direct near-field irradiation, constant average PD, and a 50% absorption factor (α = 0.5).

Total deposited energy increases linearly with exposure time, while energy distribution remains depth-dependent due to exponential attenuation. At shorter irradiation durations (e.g., 15 min), energy deposition is largely confined to superficial layers. Prolonged exposure (30–60 min) allows energy to reach deeper layers of the spheroid despite continued attenuation. This gradient provides a mechanistic explanation for observed layer-specific biological responses, with stronger stress responses expected in superficial cells and delayed or attenuated effects in inner layers.

### 2.3. Quantitative Energy Estimates

Using a WG antenna (see Section 4, Figures 10–14 and Table 2), MMW irradiation was delivered at power levels of 0.38–0.63 mW, corresponding to an average PD of 0.6 mW/cm^2^. Assuming a constant absorption factor, the estimated deposited energy increased linearly with irradiation time, while the equivalent power remained constant. Table 1 summarizes the estimated energy gradient from the spheroid surface to its base for each spheroid size and exposure duration. For spheroids with a radius of 1.68 mm, deposited energy ranged from ~942–1566 mJ after 15 min to ~3768–6264 mJ after 60 min. For larger spheroids (2.08 mm radius), energy deposition ranged from ~1494–4158 mJ after 15 min to ~5976–16,632 mJ after 60 min. These values reflect the expected increase in total absorbed energy durations. Importantly, energy deposition was non-uniform, with surface-facing cells receiving the highest exposure and deeper layers experiencing attenuated but measurable doses. This depth-dependent distribution is consistent with exponential attenuation of MMW energy in biological media. The quantitative estimates provided here describe the physical characteristics of energy delivery within the 3D spheroid model.

### 2.4. Effect of Cell Density on Cell Viability After MMW Irradiation

To optimize the cell density required for effective MMW irradiation, the NCl-H1299 (NSCLC cells) were exposed at three different cell densities (i.e., 5.0 × 10^3^ cells/10 µL, 1.0 × 10^4^ cells/10 µL, and 2.0 × 10^4^ cells/10 µL) using a WG antenna. The WG antenna provided highly localized irradiation (aperture = 4.37 mm^2^) across a broad frequency range (75–110 GHz) with an average PD of ~0.6 mW/cm^2^. Following the irradiation, the biological effects were assessed using XTT (day 2) and colony formation assay (CFA, day 10) to evaluate both acute and long-term impacts on cell proliferation and survival. Although viability, clonogenic survival, apoptosis, and senescence were quantified under 2D conditions, during MMW irradiation within the 3D spheroid-like model, consistent with the hybrid 3D-exposure/2D-analysis strategy described in Section 4.

Positive controls (doxorubicin-treated) and negative controls (sham-irradiated) were included in all experiments. MMW exposure was conducted on 3D spheroid-like aggregates, after which cells were reseeded into 2D culture for quantitative assays. This workflow ensured reproducibility while preserving the biological effects initiated during 3D exposure.

**Figure 3:**
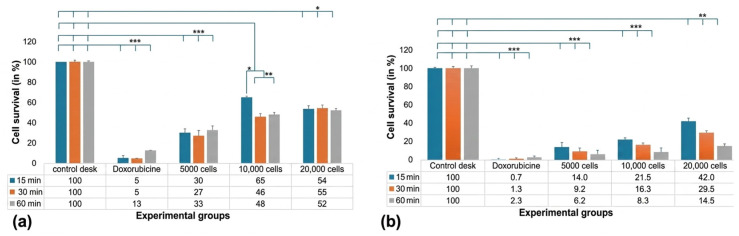
(**a**) Acute effects of MMW irradiation on NCl-H1299 cells measured on Day 2 using the XXT assay. (**b**) Long-term effects were measured on Day 10 using the colony formation assay (CFA). Each bar represents the mean value, and error bars represent the standard deviation from four biologically independent experiments (*n* = 4). Statistical analysis was performed using one-way ANOVA. Significance is indicated as follows: *p* < 0.05 (*), *p* < 0.01 (**), and *p* < 0.001 (***), comparing irradiated groups to control/sham groups.

Figure 3 presents the biological effects of non-thermal MMW irradiation on NCI-H1299 cells. These optimization experiments were performed using NCI-H1299 as the representative NSCLC model. On day 2, the highest cell survival (65%) was observed at a density of 1.0 × 10^4^ cells following 15 min of irradiation. This was followed by survival at 2.0 × 10^4^ cells after both 15- and 30 min exposures. The lowest survival (27%) occurred at 5.0 × 10^3^ cells after 30 min of irradiation. On day 10, the highest survival (42%) was observed at 2.0 × 10^4^ cells/10 µL following 15 min of exposure, whereas the lowest survival (6.2%) occurred at 5.0 × 10^3^ cells/10 µL after 60 min of irradiation. Cell viability measurements were normalized to sham-handled, non-irradiated spheroids (defined as 100% viability).

A comparison between desk-control and incubator-control spheroids (Supplementary Figure S1) showed 100 ± 0.7% viability for desk controls and 105 ± 1.2% for incubator controls (*n* = 3), indicating that handling outside the incubator did not introduce measurable stress. These controls confirm that observed differences reflect MMW-induced effects rather than artifacts of spheroid formation or handling.

The finding in Figure 3 shows that cell density in 3D droplets influences NCI-H1299 viability following MMW irradiation. Optimal survival was observed at a density of 1.0 × 10^4^ cells/10 µL following short-term exposure (15 min), suggesting that intermediate cell densities may enhance resistance to MMW-induced stress. In contrast, lower densities (5.0 × 10^3^ cells/10 µL) exhibited greater sensitivity, particularly under prolonged exposure, as evidenced by a significant reduction in cell survival on both day 2 and day 10 (see Figure 3 and Figure S4). In the subsequent experiment, cells at a lower density of 5.0 × 10^3^ cells/10 µL were used, and irradiation was applied to a single droplet under controlled conditions. Representative colony images are provided in Supplementary Figure S3, including details regarding the colony density and morphology.

### 2.5. Comparison of Antenna Systems and Power Densities on Cell Viability

Given that tumor size can vary, we aimed to identify conditions that support a high cell number within the 3D droplet model, enabling more physiologically relevant evaluation. Four antenna-amplifier configurations were tested using NCl-H1299 cells in 3D spheroid-like droplets (see Section 4, Table 2, and Figures 10–14).

**Figure 4:**
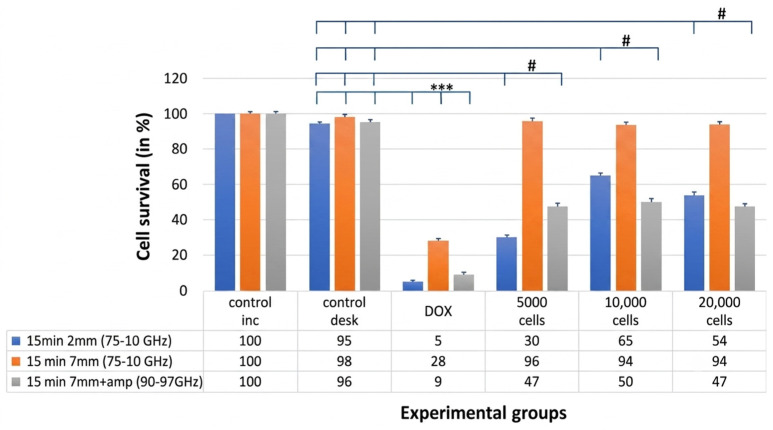
Quantification of the measured cellular response across six experimental groups. The incubator control represents cells maintained in the incubator throughout the experiment (negative control). The table control (sham control) represents cells transported and kept outside the incubator during the irradiation period. Doxorubicin (DOX)-treated cells served as the positive control. Cells were irradiated for 15 min under three exposure conditions: WG probe (2.5 × 1.75 mm; aperture 4.37 mm^2^) at 75–110 GHz (0.975 mW; blue bars); PH antenna (7 × 4.7 mm; aperture 32.9 mm^2^) at 75-110 GHz with attenuation (0.38–0.08 mW; orange bars); and PH antenna at 90–96 GHz without attenuation (4.8 mW; grey bars). Data are presented as percentages relative to the control groups. Error bars represent the standard deviation from four biologically independent experiments (*n* = 4). Statistical analysis was performed using one-way ANOVA, with significance thresholds defined as # *p* < 0.05 and *** *p* < 0.001.

Acute effects measured on day 2 post-irradiation revealed 50–70% reductions in cell survival for both the WG antenna (75–100 GHz) and the PH antenna with a multiplier without attenuation (90–96 GHz). The strongest cytotoxic effect occurred in droplets containing 5.0 × 10^3^ cells/10 µL (Figure 4). These optimization experiments were performed in NCl-H1299 cells as a representative of the NSCLC model. A key limitation of the WG antenna is its restricted irradiation area (aperture area: 4.37 mm^2^), whereas the PH antenna enables irradiation of an approximately eightfold larger area (32.9 mm^2^, see Section 4, Table 2), enabling treatment of a greater number of cells within the 3D droplet.

Several PH antenna configurations were further evaluated. Exposure using the PH antenna (75–110 GHz) with a frequency multiplier at a low average PD (~0.08 mW/cm^2^), see Section 4, Figures 10–14, and Table 2, did not significantly affect viability. In contrast, only the PH antenna with a multiplier without attenuation (90–96 GHz), delivering a ~10-fold higher PD (4.9 mW/cm^2^), produced a pronounced cytotoxic response in NCI-H1299 cells (Figure 4).

Similarly, PH antenna exposure at 90–96 GHz with attenuation generated a comparable PD (~0.08 mW/cm^2^) and did not significantly affect viability. In contrast, only the PH antenna with a multiplier without attenuation (90–96 GHz), delivering a ~10-fold higher PD (4.9 mW/cm^2^), produced a pronounced cytotoxic response in NCI-H1299 cells (Figure 4).

Long-term survival, assessed using the CFA, showed a marked reduction in clonogenic potential at a cell density of 5.0 × 10^3^ cells/10 µL, with only 10 ± 0.61%, 17 ± 1.5%, and 33 ± 2.1% of cells forming colonies after 10 days of irradiation (*p* < 0.05). Baseline viability remained consistent across matched exposure times (15, 30, and 60 min) and during screening experiments for cell density (Supplementary Figure S2). Desk-control and incubator-control comparisons (Supplementary Figure S2) showed stable viability across conditions, confirming that neither spheroid formation nor handling outside the incubator introduced measurable stress.

Overall, these results demonstrate that antenna configuration, frequency range, and attenuation significantly influence the cytotoxic response of NCI-H1299 3D tumor-like models, particularly at higher initial cell densities. Figure 4 illustrates how variations in frequency range, PD, and attenuation (mentioned in Table 2) affect cellular responses across different cell densities and experimental conditions.

Significant reductions in cell viability and clonogenic potential were observed only under conditions delivering higher APD, particularly with the PH antenna without attenuation (90–96 GHz). Lower PD exposures, regardless of frequency range or irradiation area, did not produce measurable effects. These findings highlight that effective cytotoxicity in this system requires a threshold PD, with both physical parameters and initial cell density critically determining treatment efficacy.

### 2.6. MMW Irradiation Selectively Reduces NSCLC Cell Survival

The effects of MMW irradiation on the survival of 3D NSCLC cell models were evaluated using two antenna systems: a waveguide (WG) probe (2.5 × 1.75 mm, PD 0.58 mW/cm^2^) and a PH pyramid antenna with a multiplier (7 × 4.7 mm, PD 4.9 mW/cm^2^). Two NSCLC lines (NCI-H1299 and A549) and noncancerous WI-38 fibroblasts were tested using 5.0 × 10^3^–2.5 × 10^5^ cells in 3D droplets with exposure durations of 15, 30, and 60 min. Acute effects (day 2) and long-term effects (day 10) were assessed using XTT and CFA, respectively.

Two NSCLC cell lines, NCI-H1299 and A549, and non-cancerous WI-38 fibroblasts were selected to represent distinct genetic and phenotypic backgrounds. NCI-H1299 cells are p53-null and highly proliferative with an aggressive phenotype. A549 cells carry wild-type p53 and exhibit a more epithelial and differentiated phenotype with slower proliferation. WI-38 fibroblasts are diploid, with intact cell-cycle control and low baseline proliferation, modeling normal lung tissue.

**Figure 5:**
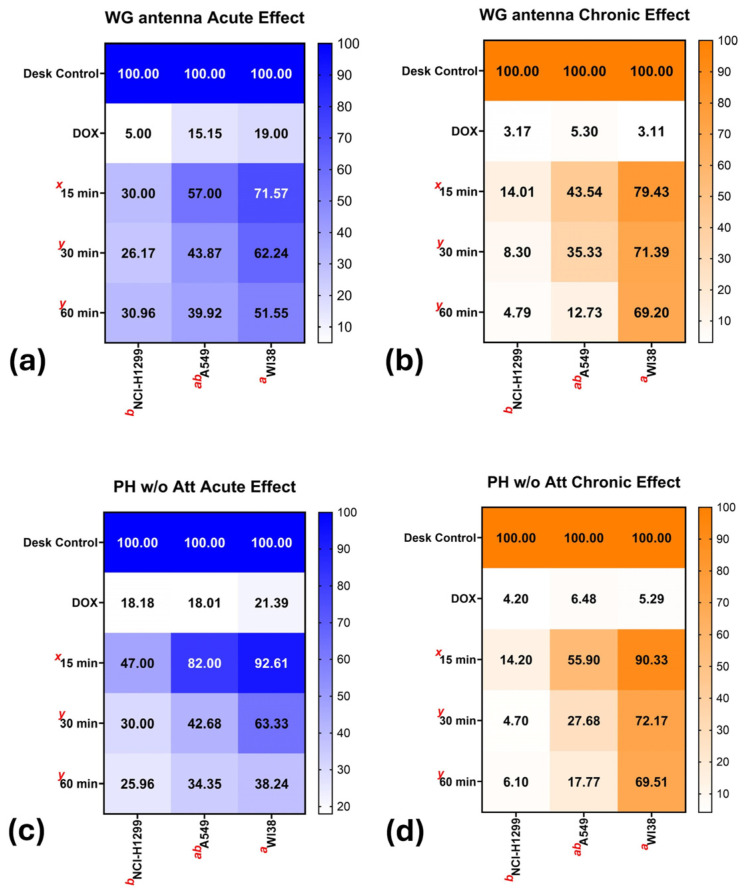
Heat map representation of acute and long-term survival responses of NCI-H1299, A549, and WI38 cells following MMW irradiation under different antenna configurations. Acute (**a**,**c**) and long-term (**b**,**d**) survival were assessed following exposure using either a waveguide (WG) antenna (2.5 × 1.75 mm; PD 0.58 mW/cm^2^) or a pyramidal horn (PH) antenna without attenuation (7 × 4.7 mm; PD 4.9 mW/cm^2^). Cell survival was normalized to sham controls (100%). Heat maps display mean ± SD values from three independent experiments (*n* = 3) and visualize the magnitude of treatment effects across exposure conditions. Statistical significance was evaluated using two-way ANOVA followed by Tukey’s post hoc test (*p* < 0.05), (see Section 4.8). Exact adjusted *p*-values are provided in Supplementary Table S1a–h.

This experimental design allows for comparison of cancer-specific versus normal-cell responses under both low- and high-density 3D conditions. The results are presented in Figure 5.

#### 2.6.1. Acute and Long-Term Effects of MMW on the NSCLC Cell Line

Figure 5 summarizes the acute (day 2) and long-term (day 10) effects of MMW irradiation delivered using WG and PH (without attenuation) antennas across NCI-H1299, A549, and WI-38 cells. Both antenna configurations induced a clear exposure time- and cell type-dependent reduction in survival, with cancer cells exhibiting greater sensitivity than non-cancerous fibroblasts. Sham control cells maintained approximately 100% survival under all conditions, whereas DOXO treatment markedly reduced survival, confirming assay sensitivity.

Under WG antenna exposure (0.58 mW/cm^2^), acute responses (Figure 5a), showed the strongest cell-type separation at 15 min, where NCI-H1299 cells demonstrated the lowest survival (30.0 ± 1.9%) compared to A549 (57.0 ± 5.03%) and WI-38 cells (71.6 ± 4.82%). Increasing irradiation time progressively reduced survival in all cell lines and diminished the differences between cell types. WI-38 cells consistently exhibited the highest acute survival, demonstrating a significant cell-type-dependent response at short exposure times.

Long-term responses (Figure 5b, Supplementary Figure S3) revealed a more pronounced long-term effect, particularly in NCI H1299 cells, where survival declined from 14.0 ± 1.0% at 15 min to 4.8 ± 0.4% at 60 min. In contrast, WI-38 cells retained substantially higher survival rates throughout the experiment (69.2 ± 1.9%) than the other tested cancer cell lines. 

The PH antenna (4.9 mW/cm^2^) produced similar but overall stronger biological effects, consistent with the higher PD used in this configuration. Acute responses (Figure 5c) again demonstrated marked cell-type differences at 15 min, with survival values of 47.0 ± 2.74% for NCI-H1299, 82.0 ± 5.03% for A549, and 93.0 ± 4.82% for WI-38. Long-term exposure further enhanced the anti-proliferative effect across all cell lines (Figure 5d, Supplementary Figure S3), while WI-38 fibroblasts maintained a relative survival advantage.

Two-way ANOVA with Tukey’s post hoc analysis revealed significant interactions between cell type and exposure duration. NCl-H1299 cells consistently formed a distinct statistical group with significantly lower survival than WI38 cells at most exposure conditions, whereas A549 cells frequently showed intermediate responses. The protective advantage observed in WI-38 cells during short exposures diminished at longer irradiation durations (30–60 min), indicating a time-dependent rather than fixed survival response. No statistical comparison was performed between antenna types because they operate at different PDs.

Overall, these findings demonstrated a selective anti-proliferative effect of MMW irradiation towards NSCLC cells, which became more pronounced under long-term exposure conditions and at higher PD irradiation. The observed reduction in long-term survival, together with increased apoptosis and senescence-associated responses, is consistent with previously reported mechanisms of MMW-induced cellular stress involving apoptosis activation and reactive oxygen species (ROS) generation [15,24].

**Figure 6:**
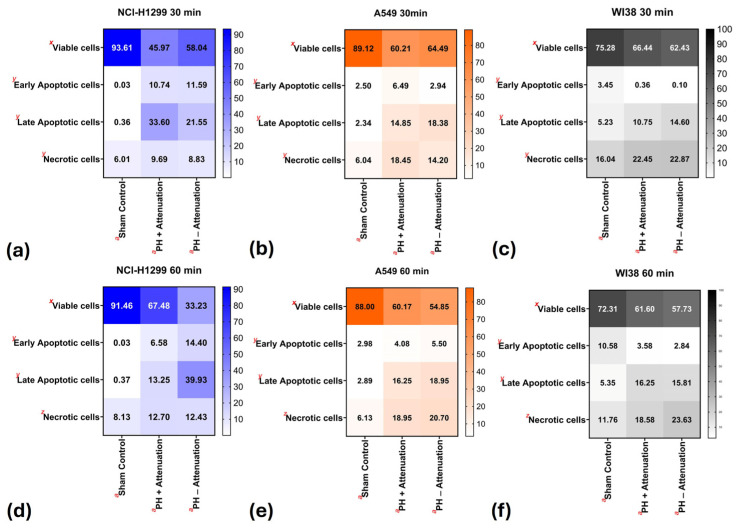
Heat map visualization of Annexin V/PI flow cytometry analysis showing the distribution of viable, early apoptotic, late apoptotic, and necrotic cell populations in NCI-H1299 (**a**,**d**), A549 (**b**,**e**), and WI38 cells (**c**,**f**) following MMW irradiation (90–96 GHz) using a 7 × 4.7 mm PH antenna. Cells were exposed for 30 min (**a**–**c**) or 60 min (**d**–**f**) under sham, PH with attenuation, and PH without attenuation conditions. Color intensity reflects shifts from viability toward apoptosis in a time- and power-dependent manner. Data represent mean percentages from three independent experiments (*n* = 3). Statistical significance was assessed using one-way ANOVA followed by Tukey’s post hoc test (*p* < 0.05) (see Section 4). Exact adjusted *p*-values are provided in Supplementary Table S2a–f.

**Figure 7:**
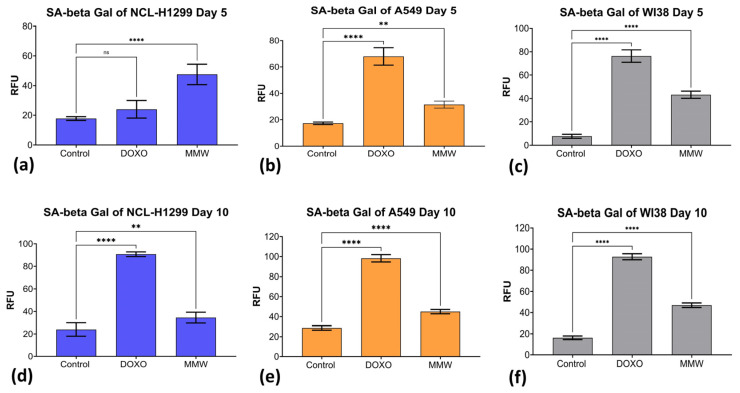
Effects of MMW irradiation on senescence-associated β-galactosidase (SA-β-gal) activity in lung cell lines NCl-H1299 (**a**) on day 5 and (**d**) day 10, for A549 (**b**) on day 5 and (**e**) day 10, and for WI38 (**c**) on day 5 and (**f**) day 10. Cells (2.5 × 10 ^5^ cells per condition) were irradiated and subsequently reseeded at 1.0 × 10^3^ cells/well in a 96-well plate. SA-β-gal activity was quantified fluorometrically and expressed as relative fluorescence units (RFU) normalized to protein content, and reflects SA-β-gal activity per unit protein. Non-irradiated cells served as negative controls, and DOXO was included as a positive control for senescence induction and assay validation. Data are presented as mean ± SD (*n* = 4). Significance is indicated as *p* < 0.01 (**), *p* < 0.001 (****) and ns, not significant (*p* > 0.05).

However, long-term survival analysis alone could not determine whether the surviving cell population retained long-term proliferative capacity or instead entered a non-proliferative senescent state. Therefore, complementary analyses were performed to distinguish between apoptotic elimination and senescence-associated growth arrest among surviving NSCLC cells. First, Annexin V/PI analysis was conducted to determine whether reduced survival was associated with apoptosis induction (Figure 6, Supplementary Figure S4). Subsequently, long-term cell survival assays (Supplementary Figure S3) together with SA-β-gal analysis (Figure 7) were performed to evaluate the proliferative potential and senescence-associated phenotype of the surviving cell population.

#### 2.6.2. Apoptotic Effect of MMW on NSCLC Cell Line

Viability and long-term survival analyses indicated that the PH antenna produced more pronounced and consistent biological effects than the WG antenna. Therefore, apoptosis analysis was performed exclusively using the PH antenna configuration.

Apoptosis was evaluated in NCl-H1299, A549, and WI-38 cells using Annexin V/PI flow cytometry (see Section 4). Heat maps were used to visualize the relative distributions of viable (Annexin-V^−^/PI^−^), early apoptotic (Annexin-V^+^/PI^−^), late apoptotic (Annexin-V^+^/PI^+^), and necrotic (Annexin-V^−^/PI^+^) populations across exposure times and irradiation conditions (Figure 6 and Supplementary Figure S4).

At 30 min of exposure, NCI-H1299 cells demonstrated a marked reduction in viable cells, together with a pronounced increase in apoptotic populations under both pH irradiation conditions. This shift was predominantly associated with late apoptosis rather than necrosis. Under the PH antenna without attenuation, viability decreased from 93.61 ± 0.55% in sham controls to 58.04 ± 4.25%, while late apoptotic cells increased from 0.36 ± 0.15% to 21.55 ± 1.41%. Similar effects were observed under attenuation conditions. In contrast, necrotic fractions remained comparatively low across all treatment groups. For the detailed dot plot segregation of the FACS analysis, readers are requested to refer to the Supplementary Figure S4.

The predominance of late apoptotic populations over necrotic fractions suggests that MMW irradiation primarily activates regulated cell death pathways rather than causing nonspecific membrane damage. This observation is consistent with previous studies reporting ROS-mediated apoptotic signaling following non-thermal MMW exposure [15,24].

A549 cells exhibited a similar but less pronounced response, with moderate increases in both early apoptotic and late apoptotic populations. In comparison, WI 38 fibroblasts retained substantially higher viability (sham control −75.28 ± 0.78%, PH antenna with attenuation 66.44 ± 1.10%, and PH antenna without attenuator −62.43 ± 1.33%) and displayed a relatively balanced distribution between apoptotic and necrotic fractions, indicating greater resistance to MMW-induced stress (see Figure 6c,f).

At 60 min exposure, these effects became more pronounced. NCI-H1299 cells exhibited a substantial loss of viability, decreasing to 33.23 ± 2.94% under PH antenna without attenuation, accompanied by a marked accumulation of late apoptotic cells (39.93 ± 1.25%). A549 cells showed intermediate sensitivity with progressive apoptotic changes (Figure 6e), whereas WI-38 cells maintained relatively higher viability and s only modest increases in apoptosis (see Figure 6f). Across all conditions, apoptotic fractions consistently exceeded necrotic fractions, supporting apoptosis as the dominant mode of MMW-induced cell death.

Tukey’s post hoc analysis demonstrated statistically significant differences among treatment conditions within specific cell death categories. PH exposure, without attenuation, significantly reduced the viable cell populations, while increasing the late apoptotic and necrotic cells compared to sham controls. These effects were most pronounced in NCl-H1299 cells and less evident in A549 and WI-38 cells. Consistent with the survival analyses, NCI-H1299 cells formed a distinct statistical group, whereas A549 cells frequently showed intermediate responses, and WI-38 cells displayed the greatest resistance to irradiation-induced apoptosis.

Overall, these findings demonstrated that prolonged MMW irradiation preferentially induces apoptosis in two NSCLC cell types, particularly in the aggressive p53-deficient NCI-H1299 cell line, while exerting limited effects on normal lung fibroblasts (WI-38). The apoptotic response increased in a time- and power-dependent manner, supporting the selective anticancer activity of MMW exposure.

To enhance the clarity and transparency of the statistical analysis, all multiple-group comparisons were supplemented with detailed pairwise comparisons using Tukey’s post hoc test. Figure 5 and Figure 6 present the statistical groupings with letter-based annotations for visual interpretation (see Section 4). Additionally, the exact adjusted *p*-values corresponding to each comparison are provided in the Supplementary File (Tables S1 and S2). These values are derived directly from the Tukey–Kramer analysis and enable a precise evaluation of statistical significance across all cell types, exposure durations, and experimental conditions.

### 2.7. MMW Irradiation Induces Senescence-Associated Growth Arrest in NSCLC Cells with Limited Effects in Normal Lung Fibroblasts

Based on the chronic survival findings in Figure 5b,d and the observed survival assay presented in Supplementary Figure S3, SA-β-gal activity was evaluated to determine whether surviving NSCLC cells underwent senescence-associated growth arrest following MMW irradiation. The analysis was performed using the PH antenna at 90–96 GHz without attenuation (4.8 mW) after 60 min of MMW irradiation, which produced the strongest anti-proliferative effects in previous experiments and enabled irradiation of a larger cell population than the WG antenna. Because chronic survival assays primarily quantify metabolically active surviving cells, they cannot distinguish proliferating cells from viable senescent cells that have lost proliferative capacity. Therefore, SA-β-gal analysis was performed to identify senescence-associated growth arrest among surviving cells. SA-β-gal activity reflects senescence-associated features in metabolically active cells undergoing growth arrest. Therefore, SA-β-gal activity was assessed on days 5 and 10 during the post-irradiation period. The results are shown in Figure 7 and Supplementary Figure S3.

MMW irradiation significantly increased senescence-associated activity in NCl-H1299, A549, and WI38 cells at both 5- and 10-day post-treatment, indicating enhanced senescence-associated responses (Figure 7a–f). Figure 5 and Supplementary Figure S3 further demonstrated that surviving NCl-H1299 cells exhibited persistently reduced proliferative recovery following irradiation, supporting the chronic anti-proliferative effects observed in Figure 5. Importantly, these experiments (Supplementary Figure S5) were performed using two initial cell densities to confirm that the observed long-term survival reduction was independent of cell density and reflected a persistent biological response to MMW exposure.

SA-β-gal activity was significantly elevated in irradiated NCl-H1299 cells (Figure 7a,d). On day 5 (post-irradiation), SA-β-gal activity increased to 47.5 ± 6.86% RFU compared with (17.8 ± 1.26% RFU) in non-irradiated controls (*p* < 0.001), indicating induction of a senescence-associated phenotype within the surviving population. Although SA-β-gal activity remained elevated until day 10 (34.5 ± 4.65% RFU, *p* < 0.01), the response was substantially lower than that induced by DOXO (90.8 ± 2.22% RFU, *p* < 0.001), suggesting partial rather than uniform induction of senescence within the surviving population.

Importantly, senescent cells remain metabolically active despite losing proliferative capacity and therefore may still contribute to chronic survival measurements. Thus, long-term survival assays alone may overestimate the fraction of cells retaining true proliferative potential after irradiation. SA-β-gal activity was measured at the population level as RFU normalized to protein content and therefore reflects relative senescence-associated activity rather than the absolute proportion of senescent cells. Consequently, no direct quantitative relationship was established between SA-β-gal activity and long-term survival measurements in the present study. Nevertheless, integration of chronic survival, cell recovery, and SA-β-gal analyses collectively supports the induction of senescence-associated growth arrest following MMW irradiation. Additionally, cell survival (Supplementary Figure S5) and SA-β-gal activity represent independent biological outcomes, and no direct quantitative relationship was established between these parameters in the present study.

Consistent with this interpretation, MMW irradiation induced similar increases in SA-β-gal activity in A549 lung cancer cells (day 5: 31.5 ± 2.65% RFU vs. 17.3 ± 0.96% RFU in control, *p* < 0.01, and day 10: 45.0 ± 2.16% RFU vs. 28.8 ± 2.22% RFU in control, *p* < 0.001 (Figure 7b,e), indicating that senescence-associated growth arrest contributes to reduced long-term proliferative capacity in NSCLC cell lines. These findings complement our apoptosis and survival results, collectively suggesting that MMW irradiation restricts cancer cell expansion through a combined effect of early cell death and delayed senescence-associated growth arrest among surviving cells.

WI-38 normal lung fibroblasts also demonstrated significantly increased SA-β-gal activity following irradiation (day 5: 43.3 ± 3.10 RFU versus 7.8 ± 1.71 RFU in controls; day 10: 47.0 ± 2.16 RFU versus 16.3 ± 1.71 RFU in controls; *p* < 0.001) (Figure 7c,f). However, unlike NSCLC cells, WI-38 fibroblasts did not exhibit pronounced chronic survival loss or marked apoptotic accumulation following irradiation. Because WI-38 cells are finite, non-immortalized fibroblasts intrinsically prone to stress- and passage-associated senescence [25,26,27], the elevated SA-β-gal activity likely reflects a stress-associated adaptive response rather than extensive cytotoxicity or irreversible proliferative collapse. This interpretation is further supported by the absence of substantial long-term cell loss compared with NSCLC cells.

Collectively, these findings demonstrate that MMW irradiation restricts long-term NSCLC expansion through a combined effect of apoptosis and senescence-associated growth arrest, while exerting comparatively weaker long-term cytotoxic effects on normal lung fibroblasts. Integration of chronic survival, cell recovery, and senescence analyses further revealed that the fraction of cells retaining true proliferative capacity after irradiation was substantially lower than suggested by metabolic survival measurements alone.

## 3. Discussion

NSCLC remains one of the most lethal malignancies worldwide, with a five-year survival rate below 20% across all stages and persistent resistance to existing therapies [1,2,4,5,25]. This study demonstrates that non-thermal MMW irradiation produces selective, PD-dependent cytotoxicity in 3D NSCLC spheroids while sparing non-cancerous fibroblasts, highlighting its translational potential as a non-invasive physical modality. Active cooling minimized bulk heating, supporting a predominantly non-thermal mechanism, although localized thermal contributions cannot be entirely excluded. By integrating physical modeling with acute and long-term biological assays, we further demonstrate that MMW-induced cytotoxicity is governed by both physical exposure parameters and tumor-specific cellular characteristics, supporting the development of MMW-based therapeutic approaches.

### 3.1. Non-Thermal MMW Irradiation in a 3D Tumor Model

The transition from 2D-monolayer cultures to 3D-tumor models represents a critical step in bridging the gap between in vitro findings and in vivo tumor behavior. 3D spheroids more faithfully recapitulate tumor architecture—including oxygen and nutrient gradients, cell–cell and cell-matrix interactions, and differential drug or radiation penetration—than conventional 2D systems [7,8,9]. The gravity-driven spheroid model used NSCLC subtypes (NCl-H1299 and A549) provides a reproducible platform for spatially resolved MMW exposure without ECM support (Figure 1). Although these structures do not fully recapitulate mature spheroids, they provide a reproducible and physically defined platform for studying spatially resolved MMW irradiation. Computational modeling confirmed that most incident W-band energy is absorbed within superficial layers (≤0.5 mm), consistent with known attenuation properties.

Previous work from our group demonstrated that MMW irradiation (75-110 GHz) using PH at relatively low PD (~0.2 mW/cm^2^) induces selective anti-cancer effects in 2D human lung cancer cells, with cancer cells showing markedly higher sensitivity than their normal counterparts [15,16]. Extending this concept to a 3D system is complex: the physical boundary conditions, energy gradients, and intercellular microenvironment fundamentally differ from those in 2D cultures [6,9]. In addition, we reported an antiproliferative, non-terminal effect using a 3D-like approach in budding yeast (*Saccharomyces cerevisiae*) by using the PH and WG antennas [17]. Specifically, wild-type *S. cerevisiae* cells exposed to MMW irradiation (85–105 GHz; ~1.0 mW/cm^2^; 50 cells/µL) for 6 h using a standard PH antenna exhibited up to a 62% reduction in division rate compared with sham controls at room temperature. Dose–response analysis further revealed that increasing power delivery via a compact WG (~17.17 mW/cm^2^) led to complete suppression of cell division after 6 h of irradiation. Temperature monitoring confirmed that these effects were non-thermal in nature. Computational modeling confirmed that most incident energy is absorbed within superficial layers (≤0.5 mm) (Figure 2) in clinically relevant temperature conditions. Here, these 2D and yeast findings are extended to NSCLC spheroids under well-controlled thermal conditions, confirming that the anti-cancer effects persist in architecturally complex 3D systems. All irradiation was performed on samples maintained at approximately 15 °C on a cooled metal platform, and temperature monitoring via a FLIR thermography camera confirmed negligible bulk heating throughout the experiments. This active cooling strategy, while important to rule out thermal artifacts, deviates from physiological temperature (37 °C); therefore, future studies will need to assess the relative contributions of thermal and non-thermal mechanisms under clinically relevant temperature conditions.

### 3.2. Integration of Physical Parameters with Biological Outcomes

A key finding of this study is that the biological response to MMW irradiation is fundamentally governed by physical parameters—most importantly, PD and antenna geometry—rather than by exposure duration alone. The current work demonstrates that the cellular response to MMW irradiation is strongly influenced by the structural context of exposure, particularly the geometry of the 3D spheroid-like droplets and the physical characteristics of the irradiation system. Both the WG and PH antenna configurations produced measurable anti-proliferative effects, but only when PD exceeded a critical threshold. Sub-threshold exposures (~0.08 mW/cm^2^ with PH under attenuation) were biologically inert across all tested cell lines and densities, while exposures at ~0.58 mW/cm^2^ (WG) and ~4.9 mW/cm^2^ (PH without attenuation) produced robust cytotoxicity. This dose-threshold behavior is broadly consistent with published data on non-ionizing electromagnetic bioeffects, where biological responses are generally negligible below a critical power level and increase non-linearly above it [26,27,28]. The recent comprehensive review by Jing et al. (2024) [14] similarly emphasizes that both the thermal and non-thermal impacts of MMW depend critically on irradiation intensity and frequency, highlighting the need for standardized protocols across studies.

Figure 3 and Figure 4 show the biological response of NSCLC cells to non-thermal MMW using a WG antenna. The WG antenna provided highly localized irradiation (aperture = 4.37 mm^2^) across 75–110 GHz with an average PD of ~0.6 mW/cm^2^ (Figures 9 and 10; Table 2). The effects were governed by both cell density within the 3D droplets and physical exposure parameters, including irradiation area, frequency range, and PD.

Cell density within the 3D droplet emerged as an additional modulator of MMW sensitivity. Figure 3 shows that cell density is a critical modulator of MMW sensitivity in the 3D tumor-like spheroid model. Lower-density droplets (5 × 10^3^ cells/10 µL) exhibited the strongest reductions in both acute viability and long-term survival, particularly under prolonged exposure. This heightened sensitivity likely reflects reduced shielding and increased per-cell energy absorption in sparsely populated droplets. In contrast, intermediate densities (1.0 × 10^4^ cells/10 µL) showed partial resistance to short-term exposure (15 min), suggesting that collective cellular organization and local microenvironmental buffering can transiently mitigate MMW-induced stress.

These findings suggest that cell density should be regarded as both an experimental and biological variable when assessing the effects of MMW in 3D tumor-like systems. The statistical analysis showed that cells had significantly different responses at 15 min compared to those at 30 min and 60 min, while no significant differences were found between the 30 min and 60 min time points. This indicates the significance of 3D tumor architecture, where factors such as cell packing, nutrient gradients, and intercellular signaling play crucial roles in stress responses. The increased vulnerability of low-density spheroids may be due to decreased paracrine support, altered dielectric properties, or higher effective energy absorption per cell. This density-dependent response likely arises from several interacting factors, including reduced shielding per cell at lower densities, modified dielectric properties of less compact aggregates, decreased paracrine survival signaling, and varying effective energy deposition per cell. Similar density-dependent effects have been observed in 3D spheroid models subjected to radiation and hyperthermia, where spatial cell packing influences heat sensitivity and clonogenic survival [29,30].

Comparative analysis of antenna systems (Figure 4, Table 2) further revealed that PD and frequency bandwidth—rather than exposure duration alone—are the primary determinants of treatment efficacy. Both WG and PH antennas at ~0.6 mW/cm^2^ and 4.8–4.9 mW/cm^2^, respectively, produced noticeable anti-proliferative effects. WG exhibited strong cytotoxicity despite the lower PD because of the highly focused irradiation field (4.37 mm^2^), whereas the PH, designed to irradiate a larger area (32.9 mm^2^), required attenuation adjustments to achieve comparable biological effects. In contrast, PH configurations operating at substantially lower PD (~0.08 mW/cm^2^)—regardless of frequency range or irradiation area—did not induce measurable cytotoxicity, indicating that MMW exposure must exceed a critical PD threshold to disrupt cellular homeostasis. Sub-threshold exposures were biologically inert under the tested conditions.

The most pronounced and consistent responses were observed with the PH antenna coupled to a frequency multiplier, which delivered a substantially higher localized PD (~4.9 mW/cm^2^) within a narrower frequency range (90–96 GHz) (Table 2; Figures 10–14). While the PH antenna enables irradiation of larger spheroids or higher cell numbers, distributed energy delivery reduces PD unless attenuation is removed. Only the unattenuated PH configuration (4.8–4.9 mW/cm^2^) produced a robust cytotoxic response, reinforcing the conclusion that PD—not antenna geometry alone—drives biological efficacy. This is consistent with our previous yeast study [17], where higher PD (17.17 mW/cm^2^) achieved complete suppression of proliferation.

Together, Figure 3 and Figure 4 demonstrate that effective MMW-based cytotoxicity requires a combination of low initial cell density, high PD, appropriate frequency ranges (75–110 GHz or 90–96 GHz), and sufficient exposure duration. These findings suggest that MMW irradiation may be particularly effective in regions of tumors with lower cellular density or disrupted architecture, such as invasive fronts or poorly vascularized zones. Conversely, densely packed tumor cores may require higher PD or prolonged exposure to achieve comparable effects. The integration of cell-density effects (Figure 3) with antenna/PD optimization (Figure 4) supports a model in which MMW-induced cytotoxicity is governed by both biological and physical thresholds. The biological threshold is defined by the structural and metabolic properties of the 3D spheroid, while the physical threshold is determined by PD and antenna characteristics. Only when both thresholds are surpassed does robust cytotoxicity occur. These analyses were performed using NCI-H1299 cells to calibrate irradiation conditions, optimize cell density, and adapt the system to the 3D model.

### 3.3. Cancer-Selective Sensitivity

One of the most clinically significant observations in this study is the preferential sensitivity of NSCLC cells to MMW irradiation compared to non-cancerous WI-38 lung fibroblasts. Under both WG and high-PD PH exposure, NCI-H1299 cells consistently showed the lowest acute and long-term survival, followed by A549 cells, while WI-38 fibroblasts maintained substantially higher viability across all conditions. Interestingly, the results shown in Figure 5 suggest that MMW irradiation exhibited cancer-selective activity across the tested lung cell line types with distinct genetic and phenotypic backgrounds. Highly proliferative, p53-deficient NCl-H1299 cells were the most sensitive, followed by p53-wild-type A549 cells, whereas normal WI-38 lung fibroblasts exhibited substantially higher survival and minimal apoptosis. Multigroup statistical analysis (Figure 5) demonstrated that the strongest separation among cell types occurred at 15 min, where WI-38 fibroblasts showed significantly higher survival than both NSCLC lines under both WG and PH exposure. At 30 min and 60 min, survival values converged, indicating that prolonged exposure reduces the relative differences among cell types. This convergence reflects a time-dependent interaction between exposure duration and cell-type susceptibility, rather than a fixed survival hierarchy.

This pattern closely mirrors the selective cytotoxicity documented in our 2D NSCLC studies [15,16] and is consistent with the general premise that cancer cells, with their elevated basal ROS, aberrant metabolism, and impaired stress-response pathways, are more vulnerable to additional electromagnetic stress than their non-cancerous counterparts [24]. Moreover, frequencies in the range of 95–105 GHz have been associated with resonance behavior of H_2_O molecules, suggesting a potential water-mediated mechanism underlying MMW bioeffects [28]. 

Figure 5 extends these observations by demonstrating cell-type–specific sensitivity across two NSCLC lines (NCI-H1299 and A549) and non-cancerous WI-38 fibroblasts. Under both WG and high-PD PH antenna exposure, NCI-H1299 consistently exhibited the lowest acute and chronic survival, followed by A549, while WI-38 fibroblasts showed the highest resistance. This selective vulnerability was most pronounced at short exposure durations and diminished with prolonged irradiation, indicating a time-dependent convergence of responses. Importantly, long-term survival remained lowest in NCI-H1299 across all conditions, supporting a selective anti-proliferative effect of MMW irradiation on cancer cells. The relative resistance of WI-38 fibroblasts suggests that normal lung cells may better tolerate MMW exposure, particularly at shorter durations.

The differential response between NCI-H1299 and A549 cells is particularly informative. NCI-H1299 cells are p53-null and highly proliferative with an aggressive phenotype, while A549 cells express wild-type p53 and display a more differentiated, epithelial phenotype with slower proliferation. The enhanced sensitivity of NCI-H1299 cells observed here—up to ~64% apoptosis at 60 min compared to lower levels in A549—is consistent with published data showing that p53-deficient tumor cells are more susceptible to DNA damage-inducing agents due to impaired checkpoint control, defective DNA repair, and failure to engage p21-mediated cell cycle arrest [31,32]. The results suggest that MMW irradiation exhibits cancer-selective activity across the tested lung cell line types with distinct genetic and phenotypic backgrounds. Highly proliferative, p53-deficient NCl-H1299 cells were the most sensitive, followed by p53-wild-type A549 cells, whereas normal WI-38 lung fibroblasts exhibited substantially higher survival. Yang et al. (2018) demonstrated that NCI-H1299 cells exhibit hypersensitivity to 8-chloro-adenosine, a DNA double-strand break-inducing agent, precisely because they lack the p53-p21 axis required for effective damage response, while A549 cells utilize this pathway to enhance repair and survival [28]. The agreement between these pharmacological findings and our MMW data suggests that MMW-induced cellular stress may converge on similar DNA-damage-related vulnerabilities. Furthermore, p53 plays a central role in the cellular response to ROS: it upregulates antioxidant genes (SESN1, SESN2) under oxidative stress conditions and coordinates apoptotic commitment via downstream targets including PUMA and BAX [33]. Cells lacking functional p53, such as NCI-H1299, are deprived of this protective axis and are therefore more prone to irreversible apoptotic progression once oxidative stress crosses a critical threshold.

In addition to p53 status, the increased basal ROS levels typical of rapidly proliferating cancer cells likely work in concert with MMW-induced oxidative stress to drive cells beyond the apoptotic threshold. This concept is supported by studies on non-thermal atmospheric pressure plasma, a type of non-ionizing physical therapy. Research has shown that this treatment preferentially induces apoptosis in cancer cells with p53 mutations through ROS-dependent pathways, while protecting normal cells that have more effective antioxidant defenses [13]. The parallels between these physical modalities—selective ROS-mediated killing of cancer cells with impaired checkpoint control—suggest shared mechanistic ground and strengthen the rationale for further mechanistic investigation of MMW irradiation.

WI-38 fibroblasts are a non-immortalized, finite diploid cell line that maintains intact cell-cycle control and has inherently lower levels of ROS. Their relative resistance to MMW exposure aligns with the broader observation that cells possessing functional p53 and significant antioxidant defenses are better equipped to handle electromagnetic stress. Importantly, the modest increase in apoptosis observed in WI-38 cells after prolonged exposure (30–60 min) indicates that these cells are not completely invulnerable; however, they remain within a tolerable dose range that cancer cells have already surpassed at earlier time points. This difference—the gap between the dose required to kill cancer cells and the dose that normal cells can tolerate—defines the therapeutic window of MMW irradiation and should be a key focus in preclinical optimization.

### 3.4. Apoptosis as the Primary Death Pathway

Annexin V/PI flow cytometry analysis confirmed that apoptosis—predominantly late apoptosis—rather than necrosis is the primary mode of MMW-induced cell death in NSCLC cells. Apoptosis assays (Figure 6) further support this selectivity. Early and late apoptotic fractions increased substantially at both 30 and 60 min exposures in NSCLC cells, while necrosis remained comparatively low, indicating that apoptosis, not necrosis, is the dominant mode of MMW-induced cell death. A549 cells showed intermediate sensitivity, whereas WI-38 fibroblasts retained higher viability and displayed only modest apoptotic changes, consistent with their relative resistance observed in Figure 5, Figure 6 and Figure 7.

Senescence analyses (Figure 7) revealed that MMW irradiation restricts cancer cell expansion through both early apoptosis and delayed senescence-associated growth arrest. NCI-H1299 and A549 cells exhibited significant reductions in long-term survival accompanied by elevated SA-β-gal activity, indicating that a substantial fraction of surviving cells entered senescence rather than resuming proliferation. WI-38 fibroblasts showed only mild SA-β-gal induction and maintained higher long-term viability. This pattern is crucial for safety reasons: apoptosis is a regulated and non-inflammatory form of cell death that does not release pro-inflammatory substances into the surrounding tissue, unlike necrosis [34]. Therefore, apoptosis induced by MMW is expected to minimize collateral inflammatory damage to healthy tissue. This presents a significant advantage over treatment methods that primarily lead to necrotic cell death.

The apoptotic response increased in a time- and PD-dependent manner across NSCLC lines, while WI-38 fibroblasts retained substantially higher viability and displayed only modest apoptotic changes at equivalent exposures. A similar pattern—dose-dependent, apoptosis-dominant cancer cell killing with sparing of normal cells—has been reported for MMW-induced apoptosis in A375 melanoma cells via caspase-3/8 activation [13], and for high-power microwave-induced intrinsic pathway apoptosis in lung cancer cells involving ROS generation and mitochondrial stress [24]. Importantly, the predominance of late apoptosis over necrosis in our data argues against simple membrane disruption as the primary mechanism and instead points toward activation of intracellular stress cascades. This is mechanistically coherent with our proposed model of ROS generation, mitochondrial dysfunction, and downstream caspase activation, described further in Section 3.5.

### 3.5. Senescence as a Secondary Anti-Proliferative Mechanism

Beyond acute apoptosis, MMW irradiation induced significant senescence-associated β-galactosidase (SA-β-gal) activity in both NSCLC cell lines, particularly on day 5 post-irradiation, indicating that a meaningful fraction of surviving cancer cells entered a state of senescence-associated growth arrest rather than resuming proliferation (Figure 5, Figure 6 and Figure 7). In NCI-H1299 cells, SA-β-gal activity increased to 47.5 ± 6.86% RFU on day 5 (vs. 17.8 ± 1.26% in controls, *p* < 0.001), and remained elevated at day 10, albeit at lower levels. A549 cells showed a similar, though somewhat less pronounced, trajectory. This dual response—early apoptosis in the most severely affected cells and delayed senescence in survivors—creates a complementary two-pronged restriction of tumor expansion.

Long-term functional viability was significantly lower than indicated by short-term assays alone. This dual response—apoptosis in highly affected cells and senescence in surviving populations—suggests that MMW irradiation disrupts tumor growth through complementary mechanisms. Normal fibroblasts exhibited only modest increases in senescence markers, consistent with a reversible stress response rather than irreversible damage. Importantly, NCI-H1299 cells exhibited significantly greater sensitivity compared to A549 cells, with higher levels of both apoptosis and long-term proliferative arrest (Figure 6). In contrast, A549 cells retained partial proliferative capacity, suggesting a more adaptive or resistant phenotype. This divergence highlights the influence of genetic background, particularly p53 status, on cellular response to MMW exposure.

The SA-β-gal response in WI-38 fibroblasts, although statistically significant, likely reflects the inherent propensity of this finite, non-immortalized cell line toward replicative and culture-associated senescence rather than a pathological response to MMW irradiation [35,36,37]. Critically, WI-38 cells did not exhibit pronounced long-term survival loss or extensive apoptotic accumulation following MMW exposure—a finding that clearly distinguishes the MMW-induced senescent response in cancer cells (associated with proliferative collapse) from the background senescence in normal fibroblasts (representing a transient, stress-adaptive state without cytotoxic consequence). This distinction underscores the cancer-selectivity of MMW irradiation and supports its safety profile at the tested PD range.

Our findings are consistent with previous reports demonstrating differential sensitivity between NSCLC cell lines with distinct genetic backgrounds. Notably, human NSCLC cell line NCI-H1299 (p53-null) cells exhibit enhanced susceptibility to DNA-damage-inducing agents compared with A549 (p53 wild-type) cells. Previous studies demonstrated that H1299 cells display hypersensitivity to 8-chloro-adenosine due to accumulation of DNA double-strand breaks, impaired DNA repair capacity, and defects in the p53–p21 signaling pathway [5,14,20,31]. In contrast, A549 cells retain more effective DNA damage response mechanisms, allowing for improved repair and survival [34,38]. These mechanistic insights are in strong agreement with our observations. In the present study, NCI-H1299 cells exhibited significantly higher levels of apoptosis (Figure 6) and long-term growth arrest (Figure 7) following MMW irradiation compared to A549 cells. The agreement between these findings and our results suggests that MMW-induced stress may converge on DNA-damage-related pathways, to which p53-deficient cells are particularly vulnerable. Thus, the enhanced sensitivity of NCI-H1299 cells likely reflects underlying DNA-repair deficiencies, supporting the central role of genetic background in determining cellular susceptibility to MMW exposure.

Importantly, these NSCLC findings are consistent with our previous work in *S. cerevisiae* [17]. The exposures were performed within ICNIRP non-thermal safety limits (20 mW/cm^2^), and temperature measurements confirmed non-thermal conditions. Comparative analysis of wild-type and *Δrad52* DNA-repair-deficient yeast strains revealed no evidence of directed genomic instability, indicating that non-ionizing MMW exposure does not induce genotoxic damage. At higher PD (17.17 mW/cm^2^), complete suppression of yeast proliferation was achieved within 3–4 h, further supporting the existence of threshold-dependent biological responses. Together, these earlier results reinforce the present NSCLC findings by demonstrating that MMW-induced growth inhibition is non-thermal, non-genotoxic, and strongly dependent on PD, exposure duration, and cellular susceptibility.

Therapy-induced senescence is increasingly recognized as a clinically relevant anti-tumor mechanism. Unlike apoptosis, senescent cells are metabolically active and viable but permanently withdraw from the cell cycle, thereby contributing to sustained tumor growth suppression [37]. In NSCLC specifically, senescence has been shown to serve as a tumor-suppressing mechanism that limits malignant transformation and proliferation [14], while senescence triggered by chemotherapy (e.g., pemetrexed) and targeted agents has been associated with improved long-term outcomes in some experimental models [16]. Our data extend this concept to non-ionizing physical therapy, demonstrating that MMW irradiation can activate a senescence program in NSCLC cells without inducing genotoxicity or thermal damage, highlighting its potential as a safe and selective anti-cancer modality.

### 3.6. Antenna-Dependent Early and Late Effects and Mechanistic Interpretation

**Figure 8:**
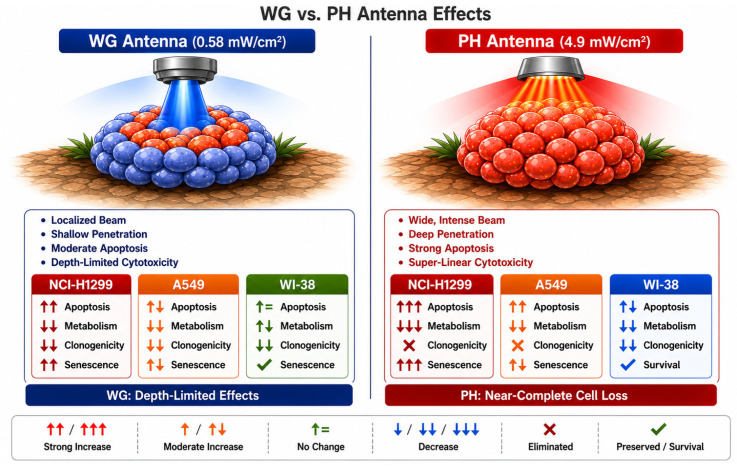
Antenna-dependent differences in power density and biological response. Comparison of WG and PH antenna configurations demonstrates that biological outcomes are governed primarily by power density rather than exposure duration. WG irradiation (0.58 mW/cm^2^) produces a localized, shallow-penetrating beam that induces moderate apoptosis and depth-limited cytotoxicity in NSCLC spheroids. PH irradiation (4.9 mW/cm^2^) delivers a wider, more intense beam with deeper penetration, resulting in strong apoptosis and super-linear cytotoxicity. The accompanying table summarizes apoptosis, metabolic activity, clonogenic survival, and senescence across three cell lines using directional symbols (↑/↓) to indicate relative changes. Cancer spheroids (NCI-H1299, A549) exhibit pronounced cytotoxicity under PH exposure, whereas normal fibroblasts (WI-38) maintain membrane integrity and survival, highlighting the selective vulnerability of tumor cells to high-PD MMW irradiation.

Figure 8 provides a comparative framework for interpreting how antenna geometry and power density shape biological outcomes under non-thermal MMW exposure. The WG antenna, with a small aperture of 4.37 mm^2^ and a peak PD of 0.58 mW/cm^2^, delivers a localized and shallow-penetrating beam. This configuration produces moderate but consistent anti-proliferative effects and induces senescence, which is consistent with a gradual accumulation of non-thermal stress over time.

In contrast, the PH antenna irradiates a substantially larger area (32.9 mm^2^) and, when driven without attenuation, delivers a nearly tenfold higher PD (~4.9 mW/cm^2^) across the 90–96 GHz range. This higher-intensity, broader beam produces pronounced early apoptosis, rapid metabolic suppression, and near-complete long-term loss of clonogenic potential. These antenna-dependent differences suggest that biological outcome is governed primarily by effective PD and spatial energy distribution rather than exposure duration alone.

The table embedded within Figure 8 summarizes these trends across three cell lines using directional symbols to indicate relative changes in apoptosis, metabolism, clonogenicity, and senescence. Cancer spheroids (NCI-H1299, A549) exhibit strong cytotoxicity under PH exposure, whereas normal fibroblasts (WI-38) maintain survival, demonstrating a clear differential sensitivity between malignant and non-malignant cells. Collectively, these observations indicate that PH exposure surpasses a critical biological threshold, producing super-linear cytotoxicity, while WG exposure induces more gradual, cumulative effects.

**Figure 9:**
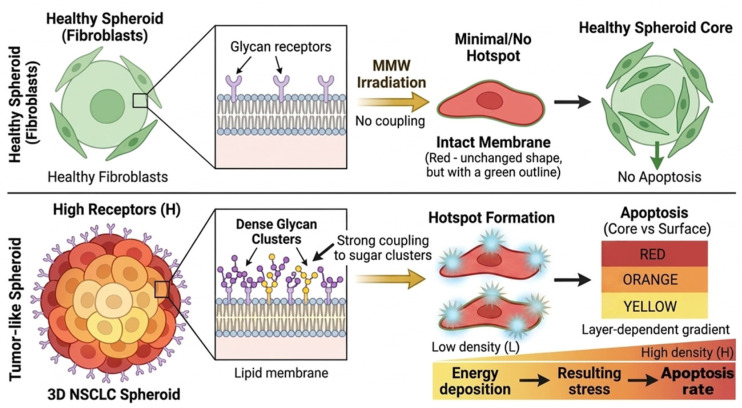
Differential coupling of MMW energy in healthy versus tumor-like spheroids. This schematic illustrates how millimeter-wave (MMW) energy interacts differently with healthy fibroblast spheroids and tumor-like NSCLC spheroids under non-thermal exposure. (1) Healthy fibroblasts exhibit low receptor density and uniform membrane organization, resulting in minimal electromagnetic coupling, preserved membrane integrity, and negligible apoptosis. (2) Tumor-like spheroids display high densities of glycan-rich, receptor-dense membrane domains that strongly couple to incident MMW energy, generating localized electromagnetic “hotspots.” (3) Hotspot formation induces nano-scale membrane disruptions, facilitating calcium influx and initiating intracellular stress signaling. (4) Superficial tumor cell layers receive the highest energy deposition, leading to ROS generation, mitochondrial dysfunction, and DNA damage. (5) As energy attenuates with depth, intracellular stress decreases, producing a layer-dependent gradient of apoptosis from the spheroid surface toward the core. The contrast between intact fibroblast membranes and receptor-dense tumor membranes highlights the cancer-selective nature of MMW-induced cytotoxicity.

To explain why PH irradiation produces rapid, high-magnitude cytotoxicity while WG irradiation yields slower, depth-limited effects, we next developed a depth- and receptor-mediated mechanistic model (Figure 9).

MMW energy forms a steep gradient within the spheroid, with the highest absorption at the surface. At the cellular level, energy preferentially interacts with glycosylated membrane domains, potentially creating localized electromagnetic “hotspots.” These regions may undergo transient nanoscale perturbations, increasing membrane permeability and enabling calcium influx [26,27,38]. This initiates intracellular stress cascades, including ROS generation, mitochondrial dysfunction, and DNA damage [36,37,38,39]. Although these pathways are biologically plausible and supported by previous studies, they were not directly measured in the present work and should therefore be regarded as mechanistic hypotheses requiring experimental validation. Cancer cells—characterized by elevated basal ROS, altered membrane composition, and impaired stress responses—are more susceptible to cumulative damage. In particular, p53-deficient cells lack efficient checkpoint control, enhancing sensitivity to sustained electromagnetic stress [17,33]. The depth-dependent attenuation of energy explains the layer-specific progression of damage, with apoptosis initiating in superficial layers and extending inward during prolonged exposure. This cumulative, non-thermal stress model aligns with previous reports demonstrating time-dependent biological effects of MMW irradiation without measurable heating [15,28].

The potential role of p53 in mediating cellular responses requires further investigation. In this study, we used NSCLC cell lines with different p53 statuses—A549 (p53 wild-type) and NCl-H1299 (p53-null)—providing an initial basis for interpreting genotype-dependent differences. However, we did not directly assess p53 involvement using targeted approaches such as gene silencing or pharmacological inhibition. Future studies will focus on elucidating the mechanistic contribution of p53 to MMW-induced stress responses.

This model is consistent with previous reports describing time-dependent, non-thermal biological effects of MMW irradiation in the absence of measurable bulk heating [15,28], and with the broader literature on non-ionizing physical modalities that exploit cancer-specific vulnerabilities—elevated ROS, altered membrane composition, impaired DNA repair—to achieve selective cytotoxicity [24,26]. It is important to note that this model remains largely mechanistic and inferential at this stage. Direct molecular evidence for calcium influx, mitochondrial dysfunction, and DNA damage following MMW exposure in our 3D system was not obtained in the current study. Future work employing targeted molecular assays—including calcium imaging, mitochondrial membrane potential measurement, γH2AX foci quantification, and ROS detection—will be essential to validate each step of the proposed pathway. Similarly, the mechanistic contribution of p53 status to MMW sensitivity should be directly assessed using isogenic cell systems with p53 gene manipulation.

Collectively, these findings suggest that non-ionizing, non-thermal MMW irradiation can selectively suppress NSCLC cells in 3D tumor-like models through spatially resolved electromagnetic interactions, without inducing comparable damage in normal lung fibroblasts. The convergence of density-dependent sensitivity, antenna-specific energy delivery, genotype-dependent susceptibility, and long-term senescence induction supports the potential of MMW irradiation as a precise, non-invasive anti-cancer strategy. Importantly, these results provide a framework for optimizing irradiation parameters in future translational studies, emphasizing that biological outcome is determined not by exposure time alone, but by the integrated physical and cellular context.

## 4. Materials and Methods

### 4.1. Cell Culture

Human NSCLC cell lines NCI-H1299 and A549, along with non-cancerous human lung fibroblasts WI-38 (ATCC CCL-75™), were obtained from the American Type Culture Collection (ATCC, IMBH for Life Science, Beit Haemek, Israel). NCI-H1299 is an aggressive lung carcinoma cell line lacking functional TP53, while A549 is a less aggressive lung adenocarcinoma line expressing wild-type TP53 and harboring KRAS mutations.

Cells were cultured as follows: NCI-H1299: RPMI-1640 medium (Gibco, Thermo Fisher Scientific, Waltham, MA, USA) supplemented with 10% fetal bovine serum (FBS), 2 mM L-glutamine, and 1% penicillin/streptomycin (Sigma-Aldrich, St. Louis, MO, USA). A549: Dulbecco’s Modified Eagle’s Medium (DMEM; Gibco) with 10% FBS, 2 mM L-glutamine, and 1% penicillin/streptomycin. WI-38: Minimum Essential Medium Alpha Modification (MEM; HyClone, Thermo Scientific) supplemented with 10% FBS. Cells were seeded in 96-well plates at 2 × 10^4^ cells per 100 µL or in 6-well plates at 1 × 10^6^ cells per 2 mL for subsequent assays.

### 4.2. MMW Irradiation Set-Up and 3D Model of Study

**Figure 10:**
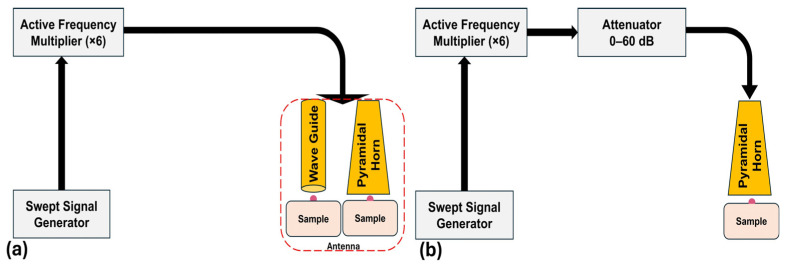
Schematic representation of the MMW irradiation setup. (**a**) The system comprises a swept signal generator (10 MHz–20 GHz; Agilent 83632B) feeding a solid-state active multiplier (Quinstar QMM-940615060; ×6), connected to one of two antenna types with distinct aperture sizes: a waveguide probe (2.5 × 1.75 mm; aperture 4.37 mm^2^) or a pyramidal horn (7 × 4.75 mm; aperture 32.9 mm^2^). This configuration generates an output frequency range of 75–110 GHz, corresponding to wavelengths (λ) of 4.0–2.725 mm. (**b**) The system includes a swept signal generator connected to the same solid-state active multiplier (×6) and coupled via an attenuator to a pyramidal horn (PH), enabling precise regulation of MMW power. This configuration operates within a narrower frequency range of 90–96 GHz, with and without attenuation.

The experimental setup was based on a W-band source feeding two types of radiating apparatus: a pyramidal horn (PH) antenna and an open waveguide (WG) probe, as schematically shown in Figure 10, with irradiation conditions summarized in Table 2. A signal generator (Agilent 83632B) producing a tunable RF signal at 10 MHz–20 GHz is followed by an x6 frequency multiplier (Quinstar QMM-940615060), producing a MMW radiation at W-band (75–110 GHz). A variable 0–60 dB attenuator (Quinstar QAD-W00000) was employed to control the MMW radiated power.

The emitted radiation was delivered to the cells via either a WG probe (2.5 × 1.75 mm; aperture 4.37 mm^2^) or a PH antenna (7 × 4.75 mm; aperture 32.9 mm^2^). The WG probe provided highly localized irradiation with relatively high-PD across the 75–110 GHz range, whereas the PH antenna enabled irradiation over a substantially larger area but at lower baseline PD.

To enhance the power output of the PH configuration, the system was optimized by operating within a narrower frequency range (90–96GHz) and by incorporating an attenuator, as shown in Figure 10b. The frequency was linearly swept between 90 GHz and 96 GHz. The scanning was continuous, using a saw-tooth driven RF signal generator (the Agilent 83623B), producing a linear ‘chirp’ between 15GHz and 16GHz with a constant amplitude. The resulting signal was then multiplied by an active frequency multiplier (×6). The sweep was repetitive with a temporal period of 100ms over the entire frequency range.

The total transmitted MMW power was measured using a Keysight M1913A power meter equipped with a Keysight W8486A power sensor. The measured average power was 16dBm (40mW) and remained constant throughout the entire frequency sweep.

**Figure 11:**
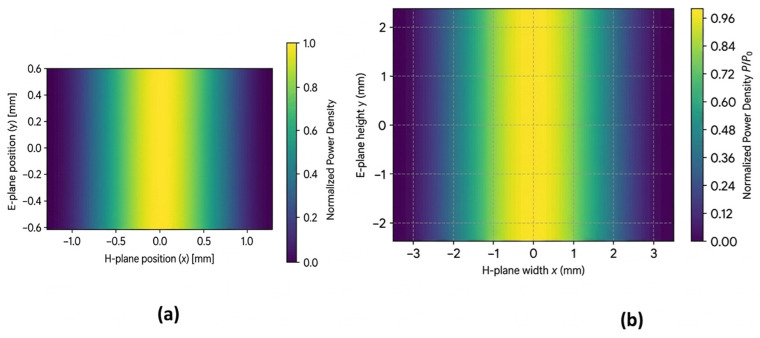
Simulated normalized power density (PD) distributions of the TE_10_ mode at the aperture of a (**a**) rectangular waveguide probe and (**b**) pyramidal horn (PH). The simulations illustrate the normalized transverse field distributions associated with the two antenna configurations.

Figure 11 presents simulations of the transverse power distribution of the radiated MMW field at the exit of the TE_10_ waveguide probe (Figure 11a) and at the aperture of the PH (Figure 11b). As shown in Figure 11a, the radiation pattern of the WG probe is too narrow to adequately illuminate the sample. Therefore, the PH was selected because its broader radiation profile better matches the sample dimensions and provides more uniform exposure. An effective average PD was estimated from the measured transmitted power and the corresponding effective exposure area.

The sample was positioned 1mm from the transmitting antenna. We note that the incident MMW radiation was maintained at a constant power level throughout the entire frequency sweep. Consequently, the PD remained constant, with no significant variation across the investigated frequency range.

**Figure 12:**
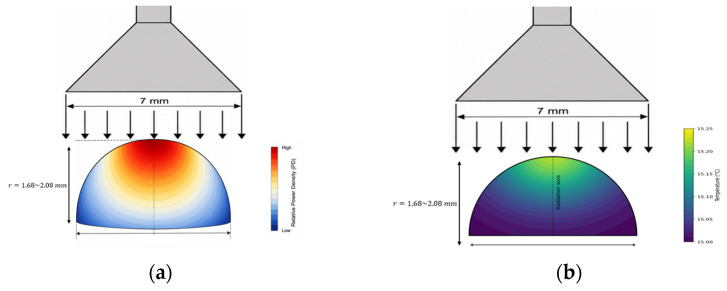
Simulation of the (**a**) radiation absorption as it penetrates the sample droplet and (**b**) the temperature distribution inside the droplet. When the sample it is illuminated by the Pyramidal horn, the absorbed power exhibits a centralized peak with symmetric radial attenuation, while the corresponding temperature rise remains modest (≈15.0–15.25 °C).

To evaluate the PD distribution within the sample droplet, we employ a dielectric model consisting of a hemispherical water-based droplet (see Figure 12), which serves as a representative model of the biological sample. At a temperature of 15 °C, the complex relative permittivity of water at 93 GHz is approximately (real part) and (imaginary part), respectively. The corresponding effective conductivity is then 50–60 s/min. It should be noted that the exposure frequency range was 90–96 GHz. Over this relatively narrow frequency band, the dielectric properties of water vary only slightly. Therefore, the dielectric parameters evaluated at 93 GHz can be considered representative of the entire exposure range.

The penetration depth of W-band MMW radiation in water-rich biological media is approximately 0.2–0.4 mm, which is significantly smaller than the sample thickness. Consequently, nearly all of the incident electromagnetic energy is absorbed within the illuminated droplet. Figure 12 presents a simulation of the PD distribution within the sample when it is illuminated by the PH, illustrating the progressive attenuation of the electromagnetic power intensity as the radiation propagates into the medium (Figure 12a), together with the corresponding temperature distribution. It should be noted that the sample was actively cooled, resulting in an average temperature of approximately 15 °C. As shown in Figure 12b, no significant temperature increase is observed throughout the exposed volume.

**Figure 13:**
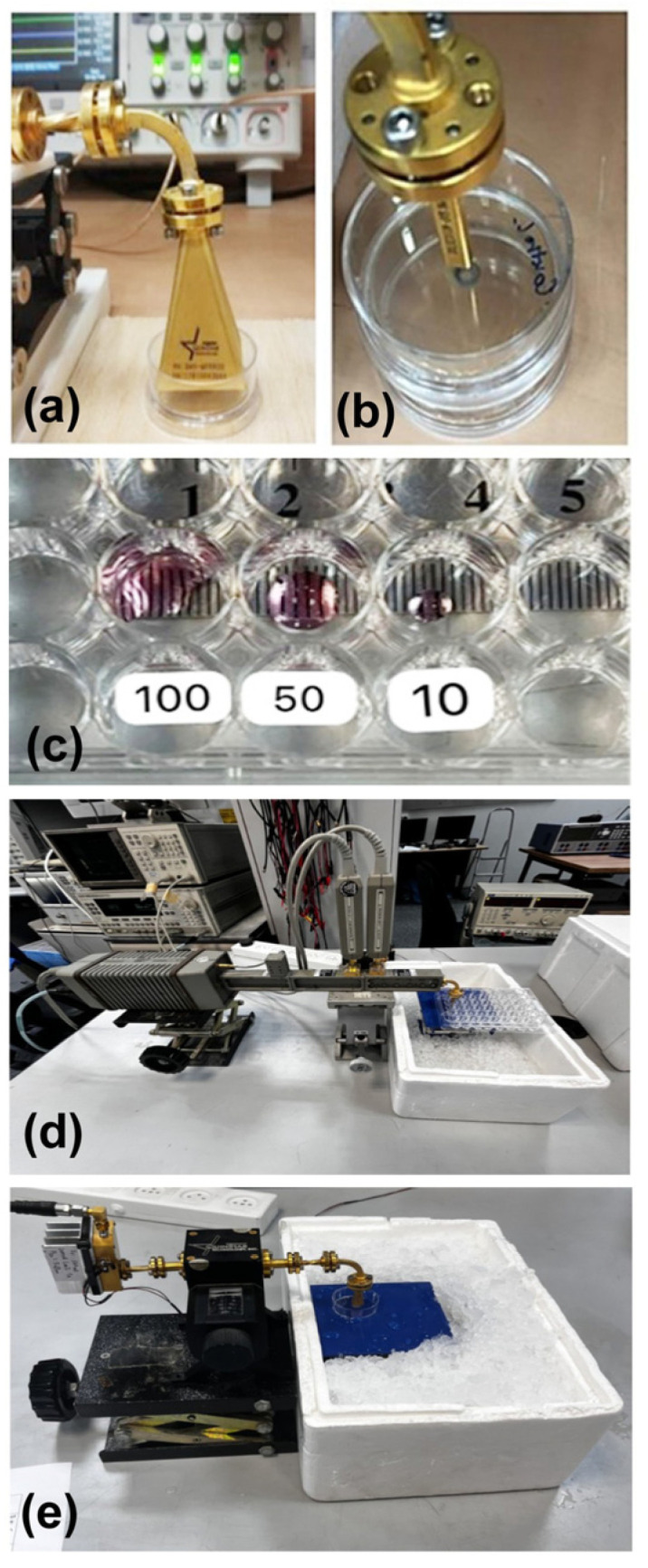
Representative experimental exposure setup images: (**a**) PH antenna; (**b**) WG antenna; (**c**) Cancer cell droplets (5 × 10^3^ cells/10 μL–2.5 × 10^5^ cells/20 μL); (**d**,**e**) Irradiation layout showing plates on ice (4–6 °C).

**Figure 14:**
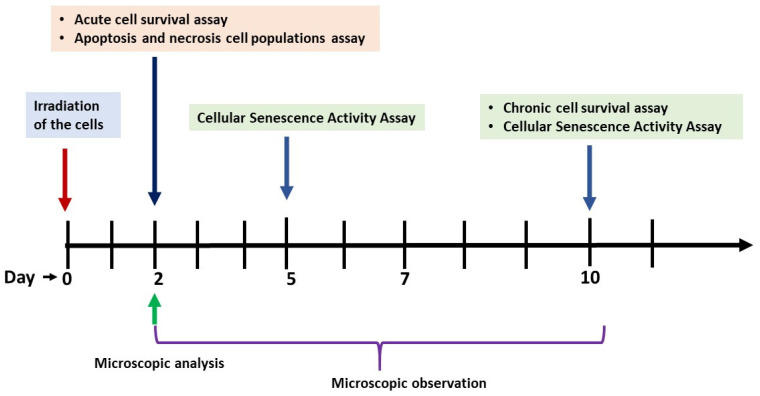
Overview of experimental schedule: acute effects (Day 2) were assessed using XTT and FACS apoptosis assays; long-term effects (Days 10–12) were evaluated by colony formation assays (CFA), measurement of senescence-associated β-galactosidase (SA-β-gal) activity, and microscopy.

Using the same setup without attenuation, the PD increased from 0.076 mW/cm^2^ to 4.9 mW/cm^2^. Due to its pyramidal geometry, the PH antenna produced a non-uniform spatial power distribution, with maximal intensity at the center and gradual attenuation toward the periphery. The cell irradiation setup and experimental schedule are presented in Figure 13 and Figure 14. Cells were detached and resuspended as small-volume droplets (10–20 µL; 5 × 10^3^–2.5 × 10^5^ cells) placed at the center of sterile 35 mm Petri dishes to form 3D cell clusters mimicking 3D-tumor architecture (3D tumor-like structure), Figure 13c. Following the droplet formation, cells were allowed to undergo rapid self-assembly within the hemispherical droplet before irradiation. This process was driven by gravitational setting and did not require extended incubation or ECM-bashed support. After irradiation, the cells were collected and reseeded for downstream assays performed under standard 2D cell culture conditions. This experimental design is intentionally chosen to maintain the physiological relevance of 3D exposure, such as depth-dependent energy gradients and cell–cell interactions, while allowing for robust and quantitative assessments using standardized 2D assays. Similar studies often involve irradiation or treatment in 3D systems, followed by post-exposure clonogenic or viability assays, which are widely used in radiation biology and tumor spheroid research [29,30,40,41]. Low- and high-power, frequency-scanning MMW radiations were applied using either the PH or WG antenna (Figure 13 and Figure 14; summarized in Table 2) across the W-band (75–110 GHz) with exposure durations of 15, 30, and 60 min. In the optimized PH configuration, the frequency range was restricted to 90–96 GHz, resulting in an approximately tenfold increase in PD compared to baseline conditions. The PH antenna produced a Gaussian-like spatial distribution, with maximal intensity at the center and decreasing toward the edges, whereas the WG probe generated a confined, high-intensity focal region (Figure 10 and Figure 13a,b, and summarized Table 2). To minimize thermal effects, samples were maintained on a cooled surface (~4–6 °C) during irradiation.

To avoid unintended cross-exposure, irradiation was performed on individual samples, as illustrated in Figure 9, using 35 mm Petri dishes rather than a multi-well plate. This approach ensured that each sample was independently exposed within a defined irradiation field. Multi-well plates were utilized only for post-irradiation assays, such as the viability and senescence assays, and were not subjected to direct MMW exposure. Key specifications of both WG and PH antennas, including aperture area, power level, and PD per unit area, are summarized in Table 2.

**Table 2:** Irradiation regimes and antenna characteristics.: Summary of antenna type, aperture size and area, frequency range, power level (dBm and W), power density (mW/cm^2^), and exposure duration.

Radiation Source	Antenna Type	Antenna Size (mm)	Aperture Area (mm^2^)	Irradiation Type	Irradiation Range (GHz)	Range Power Level	Average Power Density (mW/cm^2^)	Exposure Time (min)
Swept Signal Generator + Frequency multiplier	Wage guide probe	2.5 × 1.75	4.37	Sweeping regime	75–110	−4 dBm 0.63 mW	0.576	15, 30, 60
Swept Signal Generator +Frequency multiplier	Pyramidal	7.0 × 4.7	32.9				0.076	
Swept Signal Generator + Generator +Frequency multiplier + Attenuator					90–96			
Swept Signal Generator + Frequency multiplier + with Attenuator without attenuation						16 dBm, 40 mW	4.86	

Acute effects on cell viability were assessed using the Enhanced Cell Counting Kit-8 (E-CK-A362-500, Elabscience, TX, USA). Apoptotic cell fractions were quantified using the MEBCYTO Annexin V–FITC Apoptosis Detection Kit (cat# 4700, ENCO, Petah Tikva, Israel) by FACS. Long-term effects of MMW irradiation on cell survival and proliferative capacity were evaluated using a clonogenic CFA, together with assessment of senescence-associated SA-β-gal activity. These combined approaches enable discrimination between proliferating cells and those that remain viable but undergo senescence-associated growth arrest. Low-power irradiation: W-band (75–110 GHz), tunable output up to 10 mW, exposure durations 10, 30, and 60 min. PH antenna with frequency multiplier: Narrowed the range to 90–96 GHz and increased PD tenfold compared to WG. The PH antenna produces a Gaussian energy distribution, with the highest PD at the center and a gradual decrease toward the periphery. In contrast, WG produces a focused beam restricted to a small area. To minimize nonspecific thermal effects, plates were placed on a cold table (≈15 °C) during irradiation. Irradiation effects were evaluated at two time points: acute (2 days) and long-term (10 days).

Cell samples were placed in well plates/Petri dishes (30 mm, Thermo Fisher Scientific, Rhenium Bio, Modi’in, Israel) and maintained on ice throughout the experiments to ensure stable thermal conditions. This setup enabled precise temperature regulation, maintaining the sample temperature within ±1 °C over the entire experimental period. Under these conditions, the temperature ranged between 12 °C and 13 °C.

Temperature monitoring was performed using a FLIR thermography camera (model i7), enabling non-contact measurement of the sample surface temperature. Representative images of the experimental setup from the final experiment are provided. In addition, thermal images acquired at the beginning and at the end of the experiment (total duration: 1 h) are attached to demonstrate temperature stability over time.

According to the manufacturer’s specifications, the system provides reliable temperature measurements with an accuracy of ±2% and a thermal sensitivity (Noise Equivalent Temperature Difference, N.E.T.D.) of <0.1 °C at 25 °C.

### 4.3. Cell Viability Assay

Cell viability 48 h after MMW irradiation was assessed using the Enhanced Cell Counting Kit-8 (E-CK-A362-500, Elabscience, TX, USA). Post-irradiation, 5 × 10^3^ cells were seeded in 96-well plates with 4–6 replicates per condition. Assays were performed following the manufacturer’s instructions. Positive control: DOXO (100 nM). Negative controls: (1) untreated cells (non-irradiated/sham), (2) cells handled identically but not irradiated (“handling control”). The assay measured the acute impact of MMW treatment on NSCLC cell viability. Sham-control samples (non-irradiated) were subjected to identical handling conditions, including incubation outside the incubator, and used as the reference for normalization of all tested groups.

### 4.4. Clonogenic Assay

Long-term effects of MMW irradiation on cell survival, proliferation, and colony-forming ability were assessed using a clonogenic assay. Post-irradiated cells (500–1000, depending on the cell line) were seeded into 35 mm Petri dishes. After ~10 days, when colonies were optimally grown, cells were fixed with 100% ethanol (10 min) and stained with 0.05% crystal violet. Colonies were washed with distilled water and counted to evaluate the long-term effects of MMW exposure. All experiments were performed at least four times per cell line to ensure reproducibility.

### 4.5. Microscopy and Image Processing

The effects of MMW exposure on NSCLC cell morphology were analyzed using a Nikon fluorescent microscope (Nikon Instruments Inc., Melville, NY, USA) at 200× magnification. Images were captured with a Hamamatsu digital color-cooled camera (Bridgewater, NJ, USA) and viewed using NIS Elements software, v6.8 (Nikon Instruments Inc.). For image analysis and processing, ImageJ, v1.52 (NIH, Bethesda, MD, USA) was used to quantify morphological changes, including cell size, shape, and cluster formation.

### 4.6. Flow Cytometry

Apoptosis was evaluated immediately after MMW irradiation using the MEBCYTO Annexin V–FITC Apoptosis Detection Kit (cat# 4700, ENCO, Petah Tikva, Israel) according to the manufacturer’s instructions. For each experimental condition, approximately 2.5 × 10^5^ cells were irradiated per sample under defined exposure settings (with and without frequency multiplier). Two independent irradiation experiments were performed, resulting in approximately 5.0 × 10^5^ cells processed per condition across replicates. Following irradiation, samples were collected and, where required, pooled to obtain a final working suspension of approximately 1.0 × 10^6^ cells for staining and analysis. Cells were stained with Annexin V–FITC and propidium iodide (PI) to quantify viable, early-apoptotic, late-apoptotic, and necrotic populations. Flow cytometry data acquisition was performed using a CytoFlex S flow cytometer (Beckman Coulter, Brea, CA, USA). During acquisition, a defined subset of cells (typically 10,000–50,000 events per sample) was recorded and used for downstream analysis, rather than the total number of cells in the prepared sample. FITC fluorescence was excited at 488 nm and collected at 525/40 nm, while PI fluorescence was collected at 780/60 nm following excitation at 638 nm. Data were processed using FlowJo v10.0.7 software (Treestar Inc., Ashland, OR, USA) to determine the percentage distribution of apoptotic, necrotic, and viable cells. All experiments were performed in triplicate (n = 3), and data are presented as mean ± standard deviation.

### 4.7. Cellular Senescence Activity Assay

Briefly, the cells were grown in T75 flasks until they reached 80–90% confluence. Later, the cells are detached, and the desired cell suspension density (1.25 × 10^6^ cells/20 µL) is prepared. Later, the cell suspension drop was placed in 35 mm Petri dishes, thereby forming 3D spheroid-like structures. Following irradiation, cells were collected from spheroids and seeded into a 94-well plate at a density of 1.0 × 10^3^ for assay, then incubated. The media was changed every day. On days 5 and 10, cells were harvested, and protein content (for sample normalization) was analyzed using the Pierce BCA Protein Assay Kit (23227, Thermo Fisher Scientific, USA). SA-β-galactosidase activity was then assessed using the Cellular Senescence Assay Kit (# ENZ-KIT12923227, Enzo Life Sciences, New York, NY, USA). It used the β-gal substrate 4-Methylumbelliferyl β-D-galactopyranoside (4-MUG) to detect SA-β-gal activity. Upon binding to β-gal, 4-MUG is hydrolyzed to the fluorescent product 4-MU, which can be measured at an excitation wavelength of 360 nm and an emission wavelength of 465 nm. Fluorescence intensity correlates with β-gal levels in the sample, as measured using an Infinite^®^ M200PRO multimode multiplate reader (Tecan Life Science, Tecan Group Ltd., Männedorf, Switzerland). This quantitative assay uses cellular lysates to determine SA-β-gal activity. Fluorescence signals were normalized to total protein content before data analysis and were reported as RFU.

### 4.8. Statistical Analysis

All experiments were conducted with at least three independent replicates (*n* ≥ 3–6). The data are presented as mean ± standard deviation (SD). Statistical comparisons between groups were performed using either Student’s *t*-test or one-way ANOVA, as appropriate. A *p*-value of less than 0.05 was considered statistically significant. For comparisons involving multiple groups, the Tukey–Kramer multiple-comparison test was applied. Statistical significance among groups was indicated using letter-based annotations, where groups sharing at least one letter are not significantly different from each other. In this system, annotations such as (a, b, ab) denote statistical groupings among cell types at the same exposure and conditions. Meanwhile, annotations such as (x, y, and/or z) indicate statistical groupings across different exposure durations within the same cell type and condition. Groups that share at least one letter are not significantly different, while those that do not share any letters are considered to differ significantly. Exact adjusted *p*-values for all pairwise comparisons are reported in Supplementary Tables S1 and S2. Statistical analyses were performed using JMP Pro 16, with a significance threshold of *p* < 0.05.

## 5. Conclusions

This study demonstrates that non-thermal MMW irradiation induces selective, PD-dependent cytotoxicity in 3D NSCLC spheroids while sparing normal fibroblasts. The integration of physical modeling, antenna comparison, and multi-layered biological assays reveals that the PD is the dominant determinant of efficacy, with a clear threshold separating inert from cytotoxic exposures. Cancer-selective vulnerability arises from intrinsic stress-response deficiencies, particularly in p53-null NCI-H1299 cells. Apoptosis is the primary mode of cell death, complemented by senescence-associated growth arrest. Antenna configuration shapes biological outcomes, with PH irradiation producing rapid, super-linear cytotoxicity and WG irradiation yielding depth-limited, cumulative effects. The mechanistic model supports a receptor-mediated, depth-dependent stress pathway involving membrane hotspots, mitochondrial dysfunction, and intrinsic apoptosis.

Together, these findings establish a mechanistic and experimental foundation for advancing MMW-based strategies as a non-invasive, selective anti-cancer modality. Future work should evaluate MMW effects at physiological temperature, explore combinatorial regimens, and assess in vivo relevance to further define the therapeutic window.

## Data Availability

The original contributions presented in this study are included in this article. Further inquiries can be directed to the corresponding author.

## Supplementary Materials

The following supporting information can be downloaded at: https://www.mdpi.com/article/10.3390/ijms27125621/s1.

## Abbreviations

The following abbreviations are used in this manuscript:

MEM: Minimum essential medium, µL: Microliter, mL: Milliliter, mm: Millimeter, PH: Pyramidal horn, WG: Waveguide, mW: Milliwatt, °C: Degree centigrade Celsius, h: Hours, nM: Nanomolar, XTT: 2,3-bis(2-methoxy-4-nitro-5-sulfophenyl)-5-carboxanilide-2H-tetrazolium, nm: Nanometer, ANOVA: Analysis of variance, SD: Standard deviation, cm: Centimeter, µm: Micrometer, PD: Power density, mJ: Millijoule, CFA: Colony formation assay, ROS: Reactive oxygen species.
