# Supplementary material for: Effects of Nonionizing Millimeter-Wave on Spheroid-like Irradiated Non-Small-Cell Lung Cancer (NSCLC) Cells
Source: Int J Mol Sci. 2026 Jun 22;27(12):5621. doi: 10.3390/ijms27125621 (PMC13300662; doi:10.3390/ijms27125621)
Supplement: Supplementary file 1 [file ijms-27-05621-s001.zip › ijms-4246142-supplementary.pdf]

<u>Supplementary File</u>

Effects of Nonionizing Millimeter-Wave on Spheroid-like Irradiated Non-Small-Cell Lung Cancer (NSCLC) Cells

Helena Tuchinsky [1], Boris Litvak [2], Vladimir Freydin [2], Firas Simaan [2], Rawad Said [2], Dhaval Patel [1], Yosef Pinhasi [2], Asher Yahalom [2,3,4] and Stella Liberman-Aronov [1,*]

[1] Institute of Personal Medicine, Ariel University, P.O.B. 3, Ariel 40700, Israel; helenat@ariel.ac.il (H.T.); dhaval30103@gmail.com (D.P.)
[2] Department of Electrical & Electronic Engineering, Ariel University, P.O.B. 3, Ariel 40700, Israel; borisl@ariel.ac.il (B.L.); vladimirfr@ariel.ac.il (V.F.); xrayfiras@hotmail.com (F.S.); rawad.said316@gmail.com (R.S.); yosip@ariel.ac.il (Y.P.); asya@ariel.ac.il (A.Y.)
[3] Center for Astrophysics, Geophysics, and Space Sciences (AGASS), Ariel University, P.O.B. 3, Ariel 40700, Israel
[4] FEL User Center, Ariel University, P.O.B. 3, Ariel 40700, Israel
* Correspondence: stellar@ariel.ac.il; Tel.: +972-3-937-1431

1. Control Conditions

To assess the anticancer activity of millimeter waves (MMW), two types of antenna systems–a waveguide probe (2.5 × 1.75 mm, PD 0.58 mW/cm^2) and a PH pyramid antenna with a multiplier (7 × 4.7 mm, PD 4.9 mW/cm^2)- were used to expose the NCl-H1299 cells to MMW for 15 minutes. Once the antenna system was determined, the screening of cell density for optimum anti-cancer activity using the potential antenna system was also checked. The native unirradiated cells served as the control group. The experiment included two control groups: a sham control (or control desk) and an incubator control, and three experimental replicates. During irradiation, the cells were kept outside the incubator for 15, 30, or 60 minutes. Therefore, we believe that the sham control is more appropriate than the incubation control. However, the effects of incubation inside and outside the incubator are also worth exploring. The results for both control groups are illustrated in the following figures.

1.1 Screening of Antenna

In the present experiment, the NCl-H1299 cells were exposed to MMW using the above-mentioned antenna systems. Cell viability measurements were normalized to sham-

handled, non-irradiated spheroids (desk control), which were defined as 100% viability. Under native, unirradiated conditions, baseline viability remained high, and variability was low. As shown in Figure S1, desk-control spheroids exhibited 100 ± 0.7% viability, while incubator controls showed slightly elevated metabolic activity (105 ± 1.2%, n = 3), likely reflecting the absence of handling-associated stress (see **Figure S1**).

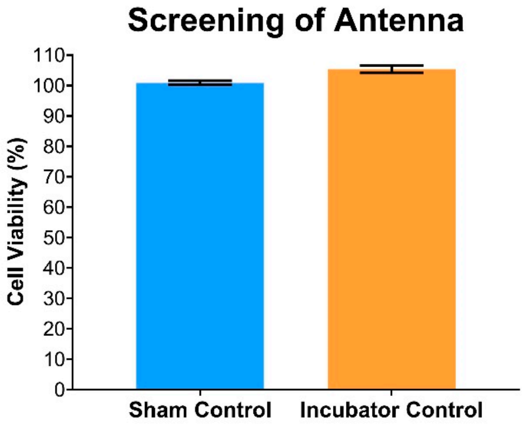

Figure S1. Baseline viability of native, unirradiated spheroids under control conditions. Cell viability was normalized to the sham-handled (desk) control, defined as 100%. Incubator controls show slightly higher metabolic activity, reflecting the absence of handling-associated stress. Data are presented as the mean ± SD of three biological replicates (n = 3).

1.2 Screening of Cell Density and Exposure Controls

To evaluate the optimal anticancer activity of the pyramidal horn antenna with attenuation, different cell densities (5×10^3–2.5×10^5 cells in 10–20 µL droplets) of NCl-H1299 cells were irradiated at three time points, i.e., 15-, 30-, and 60-min. Cell-viability measurements were normalized to sham-handled, non-irradiated spheroids (desk control), as mentioned in the following figure (**Figure S2**). Baseline viability remained stable across exposure-matched time points (15, 30, and 60 min) and during cell-density screening experiments (**Figure S2**), confirming that neither spheroid formation nor experimental handling induced significant loss of viability. These observations validate the use of the desk control as an appropriate reference for normalization of all irradiated conditions.

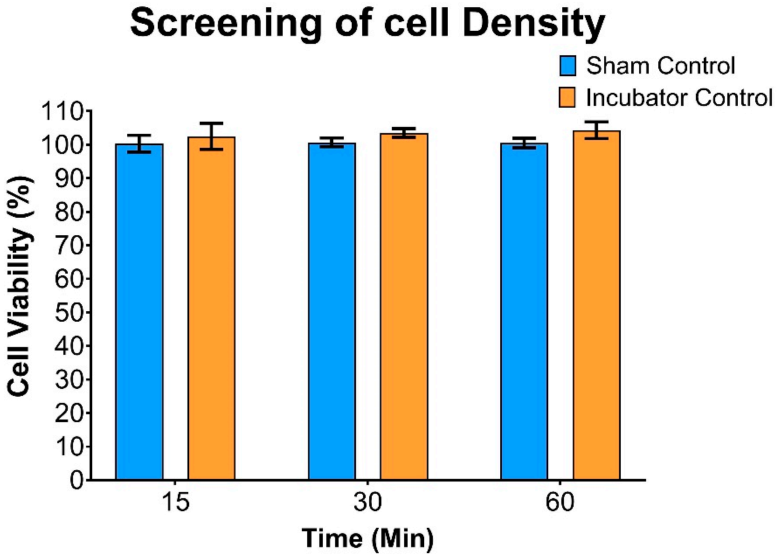

Figure S2. Baseline viability of native, unirradiated spheroids during cell-density and exposure-time screening. Sham-handled (desk), and incubator controls were monitored at 15, 30, and 60 min to match irradiation conditions. Cell viability remained stable across time points and conditions, confirming that experimental handling and incubation outside the incubator did not significantly reduce viability. Data are mean ± SD (n = 3).

2. Colony density and morphology following the MMW irradiation

This study investigates whether MMW irradiation affects not only clonogenic survival but also the appearance of cell colonies. We present representative images from colony formation assays illustrating colony density and morphology of lung cancer cell lines (NCI-H1299, A549) and non-cancerous WI-38 cells under both control conditions and MMW irradiation (**Figure S3**). The images visually support the quantitative data on clonogenic survival by showing a time-dependent reduction in colony density following irradiation, while individual colony morphology remains largely unchanged.

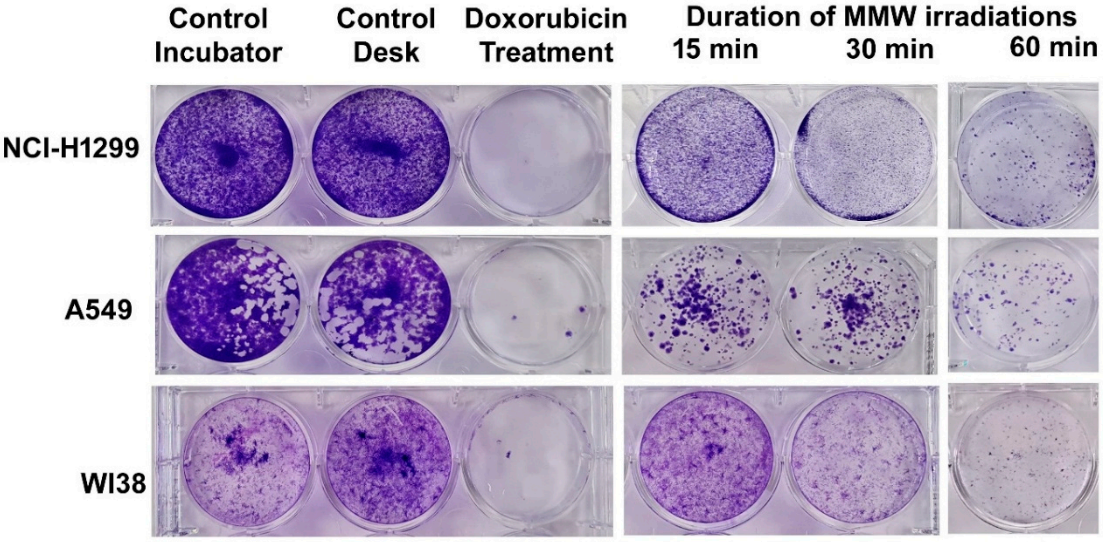

Figure S3. Representative images of CFA illustrating colony density and morphology of NCI-H1299 and A549 lung cancer cells and non-cancerous WI-38 fibroblasts following MMW irradiation. Cells were subjected to sham incubation (incubator and desk controls), doxorubicin treatment (positive control), or MMW exposure for 15, 30, and 60 min using the specified irradiation conditions. Colonies were fixed and stained with crystal violet after the growth period. MMW irradiation resulted in a time-dependent reduction in colony density in cancer cells, with progressively fewer and smaller colonies observed at longer exposure times, whereas WI-38 fibroblasts displayed comparatively preserved colony formation. No major alterations in individual colony shape were observed, indicating that MMW irradiation primarily affects clonogenic potential rather than inducing aberrant colony morphology. Quantitative analysis of the corresponding cell populations is presented in Figure 5 (b,d) of the main manuscript.

3. Pairwise statistical comparisons corresponding to Figure 5 and Figure 6

Tables S1 and S2 summarise all pairwise comparisons among NCI-H1299, A549, and WI-38 cells across various exposure durations and antenna conditions. Statistical analysis was conducted using ANOVA, followed by the Tukey–Kramer multiple comparisons test in JMP Pro 16. Adjusted p-values and their corresponding significance levels are reported for each comparison.

To facilitate interpretation of multiple group comparisons, statistical differences were summarized using letter-based annotations (as seen in Figure 5 and Figure 6) derived from post hoc analysis. Groups sharing at least one common letter (e.g., a, ab) are not significantly different from each other, whereas groups with no shared letters (e.g., 'a' vs 'b' or 'x' vs 'y' vs 'z') are significantly different at $p < 0.05$. In this scheme, a single letter (e.g., 'a' or 'b' or 'x' or 'y' or 'z') indicates membership within a distinct statistical group, while combined letters (e.g., ab) indicate that a group does not significantly differ from multiple groups that are assigned those respective letters. This approach provides a concise visual summary of pairwise comparisons while accounting for multiple testing. All annotations are based on pairwise comparisons of group means, with statistical significance determined at $\alpha = 0.05$.

Corresponding p-values, mean differences, standard errors, and confidence intervals are reported in the following tables (Tables S1 and S2). These results provide the quantitative foundation for the letter-based groupings illustrated in Figures 5 (in Table S1a-h) and Figure 6 (in Table S2a-f).

Table S1a. Ordered Differences report on cell types irradiated with the WG for acute effects.

Cell Type Comparison	Differences	Standard error of difference	Lower CL	Upper CL	p-Value (α = 0.05)
NCl-H1299 vs WI38	22.06	7.93	3.16	40.97	0.0179
NCl-H1299 vs A549	14.40	7.93	– 4.50	33.30	0.1702
A549 vs WI38	7.66	7.93	– 11.24	26.57	0.5997

Table S1b. Ordered Differences report among Irradiation Time using the WG for acute effect.

Irradiation Time Comparison	Differences	Standard error of difference	Lower CL	Upper CL	p-Value (α = 0.05)
15 vs 60	23.15	5.65	9.52	36.79	0.0004
15 vs 30	17.25	5.65	3.61	30.88	0.0099
30 vs 60	5.91	5.65	– 7.73	19.55	0.5518

Table S1c. Ordered Differences report on cell types irradiated with the WG antenna for long-term effects.

Cell Type Comparison	Differences	Standard error of difference	Lower CL	Upper CL	p-Value (α = 0.05)
NCl-H1299 vs WI38	19.18	7.82	0.53	37.83	0.0425
NCl-H1299 vs A549	11.51	7.82	– 7.14	30.16	0.3095
A549 vs WI38	7.66	7.82	– 10.99	26.15	0.5914

Table S1d. Ordered Differences report among Irradiation Time using the WG for long-term effects.

Irradiation Time Comparison	Differences	Standard error of difference	Lower CL	Upper CL	p-Value (α = 0.05)
15 vs 60	26.68	5.17	14.21	39.16	<0.001
15 vs 30	19.11	5.17	6.64	31.58	0.0015
30 vs 60	7.57	5.17	– 4.90	20.05	0.3157

Table S1e. Ordered Differences report on cell types irradiated with the PH antenna without an attenuator for acute effects.

Cell Type Comparison	Differences	Standard error of difference	Lower CL	Upper CL	p-Value (α = 0.05)
NCl-H1299 vs WI38	22.06	7.93	3.16	40.97	0.0179
NCl-H1299 vs A549	14.40	7.93	– 4.50	33.30	0.1702
A549 vs WI38	7.66	7.93	– 11.24	26.57	0.5997

Table S1f. Ordered Differences report among Irradiation Time using the PH antenna without the attenuator for acute effect.

Irradiation Time Comparison	Differences	Standard error of difference	Lower CL	Upper CL	p-Value (α = 0.05)
15 vs 60	23.15	5.65	9.52	36.79	0.0004
15 vs 30	17.25	5.65	3.61	30.88	0.0099
30 vs 60	5.91	5.65	– 7.73	19.55	0.5518

Table S1g. Ordered Differences report on cell types irradiated with the PH antenna without an attenuator for long-term effects.

Cell Type Comparison	Differences	Standard error of difference	Lower CL	Upper CL	p-Value (α = 0.05)
NCl-H1299 vs WI38	19.18	7.82	0.53	37.83	0.0425
NCl-H1299 vs A549	11.51	7.82	– 7.14	30.16	0.3095
A549 vs WI38	7.66	7.82	– 10.99	26.15	0.5914

Table S1h. Ordered Differences report among Irradiation Time using the PH antenna without the attenuator for long-term effects.

Irradiation Time Comparison	Differences	Standard error of difference	Lower CL	Upper CL	p-Value
15 vs 60	26.68	5.17	14.21	39.16	<0.001
15 vs 30	19.11	5.17	6.64	31.58	0.0015
30 vs 60	7.57	5.17	– 4.90	20.05	0.3157

Table S2a. Ordered Differences report among antenna types used to irradiate cells for 30 min with the PH antenna without an attenuator for apoptosis.

Cell Type Comparison	Differences	Standard error of difference	Lower CL	Upper CL	p-Value (α = 0.05)
Control Desk vs. PH w/o Att	3.55×10^{-15}	5.50	–13.04	13.04	1.0000
PH with Att vs. PH w/o Att	3.55×10^{-15}	5.50	–13.04	13.04	1.0000
PH with Att vs Control Desk	0	5.38	–12.75	12.75	1.0000

Table S2b. Ordered Differences report among cell stages for apoptosis after 30 min irradiation with the PH antenna without an attenuator.

Cell Stages Comparison	Differences	Standard error of difference	Lower CL	Upper CL	p-Value (α = 0.05)
Viable vs. Early Apoptotic	57.78	3.05	49.85	65.70	< 0.0001
Viable vs. Late Apoptotic	54.98	3.05	47.06	62.90	< 0.0001
Viable vs. Necrotic	54.67	3.05	46.75	62.59	<0.0001
Necrotic vs. Early Apoptotic	3.11	3.05	– 4.81	11.02	0.7381
Late Apoptotic vs Early Apoptotic	2.80	3.05	– 5.12	10.72	0.7941
Necrotic vs. Late Apoptotic	0.30	3.05	– 7.62	8.22	0.9996

Table S2c. Ordered Differences report among the cells for apoptosis after 30 min irradiation with the PH antenna without an attenuator.

Cell Type Comparison	Differences	Standard error of difference	Lower CL	Upper CL	p-Value (α = 0.05)
NCl-H1299 vs WI38	4.38	5.62	– 8.48	18.15	0.67
NCl-H1299 vs A549	4.38	5.62	– 8.48	18.15	0.67
A549 vs WI38	1.06×10^{-14}	5.62	– 13.31	13.31	1.0000

Table S2d. Ordered Differences report among antenna types used to irradiate cells for 60 min with the PH antenna without an attenuator for apoptosis.

Cell Type Comparison	Differences	Standard error of difference	Lower CL	Upper CL	p-Value (α = 0.05)
Control Desk vs. PH w/o Att	3.55×10^{-15}	5.50	–13.04	13.04	1.0000
PH with Att vs. PH w/o Att	3.55×10^{-15}	5.50	–13.04	13.04	1.0000
PH with Att vs Control Desk	0	5.38	–12.75	12.75	1.0000

Table S2e. Ordered Differences report among cell stages for apoptosis after 60 min irradiation with the PH antenna without an attenuator.

Cell Stages Comparison	Differences	Standard error of difference	Lower CL	Upper CL	p-Value (α = 0.05)
Viable vs. Early Apoptotic	60.24	2.54	53.63	66.85	< 0.0001
Viable vs. Late Apoptotic	51.81	2.54	45.19	58.42	< 0.0001
Viable vs. Necrotic	51.65	2.54	45.04	58.26	< 0.0001
Necrotic vs. Early Apoptotic	8.59	2.54	1.98	15.29	0.0052
Late Apoptotic vs Early Apoptotic	8.43	2.54	1.82	15.04	0.0063
Necrotic vs. Late Apoptotic	0.15	2.54	– 6.46	6.77	0.9999

Table S2f. Ordered Differences report among the cells for apoptosis after 60 min irradiation with the PH antenna without an attenuator.

Cell Type Comparison	Differences	Standard error of difference	Lower CL	Upper CL	p-Value (α = 0.05)
NCl-H1299 vs WI38	0	5.50	– 13.03	13.04	1.0000
NCl-H1299 vs A549	7.11×10^{-15}	5.38	– 12.75	12.75	1.0000
A549 vs WI38	7.11×10^{-15}	5.50	– 13.03	13.04	1.0000

4. Apoptosis Assay by FACS

Annexin V/PI flow cytometry analyses were conducted to evaluate the cell death profiles induced by MMW exposure in a 3D-like spheroid model. Apoptosis was assessed immediately after MMW irradiation using the MEBCYTO Annexin V–FITC Apoptosis Detection Kit (catalog #4700, ENCO). The analysis was performed on a CytoFlex S flow cytometer (Beckman Coulter, Brea, CA, USA) with a sample size of 1.0×10^6 cells per sample. The representative dot plots (**Figure S4**) illustrate the distributions of viable, early-apoptotic, late-apoptotic, and necrotic cell populations under both control and irradiation conditions. The data indicate a shift from viable cells to apoptotic populations as exposure time and power density increase, with minimal induction of necrosis observed.

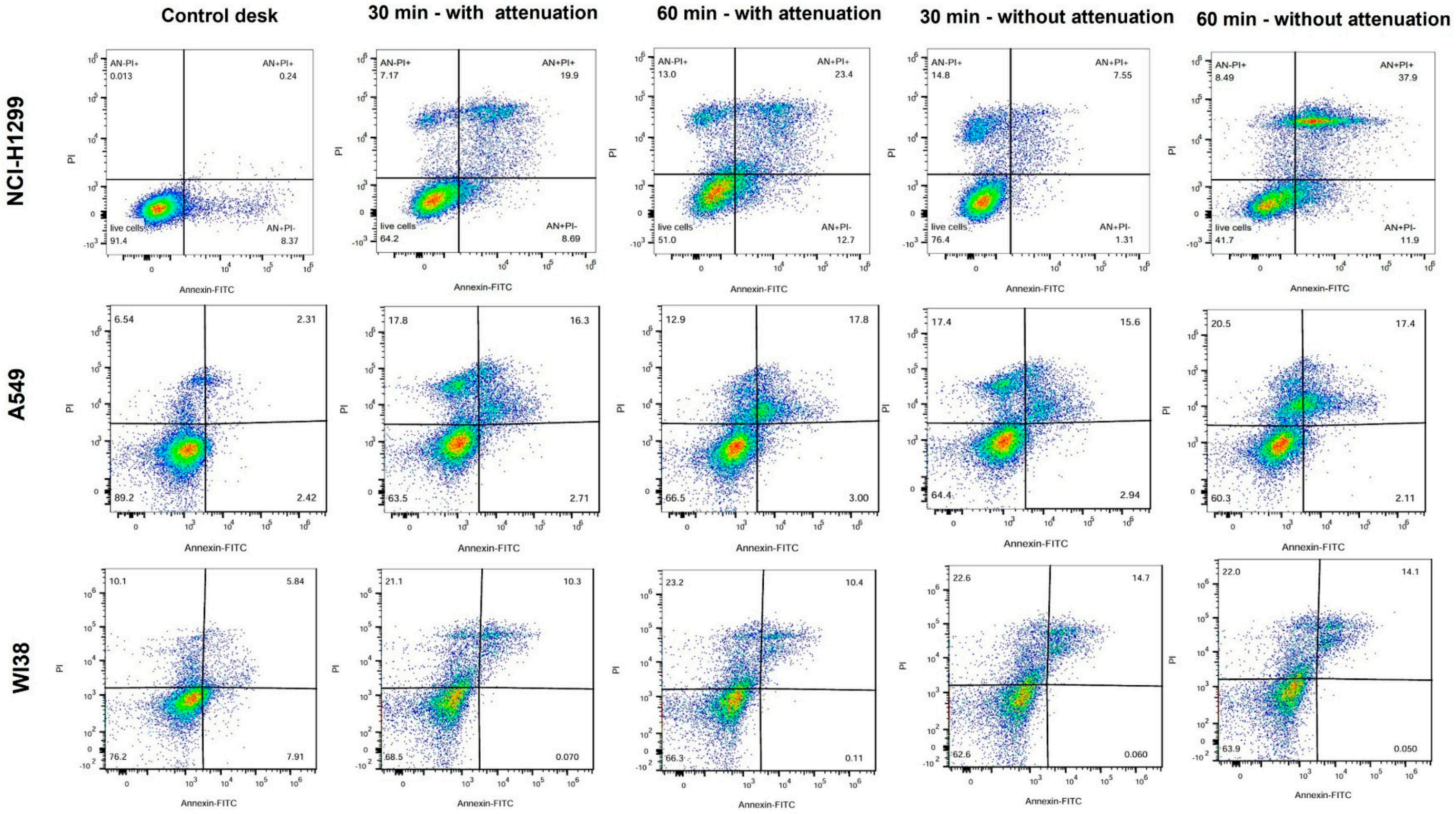

Figure S4. Representative Annexin-V/PI flow-cytometry dot plots of lung cancer cells (NCI-H1299 and A549) and non-cancerous WI-38 fibroblasts under control and MMW-irradiation conditions using the pyramidal horn (PH) antenna. Cells were stained immediately after treatment and analysed by flow cytometry. Quadrants indicate viable (Annexin-V$^-$/PI$^-$), early apoptotic (Annexin-V$^+$/PI$^-$), late apoptotic (Annexin-V$^+$/PI$^+$), and necrotic (Annexin-V$^-$/PI$^+$) populations. Representative plots are shown for sham-handled controls and for 30- and 60-min exposures with and without attenuation. Quantitative analysis of the corresponding cell populations is presented in Figure 6 of the main manuscript.

Long-term survival of viable NCl-H1299 cells following MMW irradiation

NCl-H1299 cells were exposed to millimetre-wave (MMW) irradiation using a PH antenna without attenuation, operating at 90-96 GHz and 4.8 mW, for 60 min. However, the chronic survival assay presented in Figure 5 and Supplementary Figure S3 primarily quantified the metabolically active surviving cell population using the clonogenic assay. It did not distinguish cells that retained proliferative capacity from viable cells undergoing senescence-associated growth arrest. Therefore, additional long-term survival experiments were performed. To evaluate the long-term proliferative capacity of surviving lung cancer cells following irradiation, two cell densities were examined: 2.0 × 10^3 and 5.0 × 10^3 cells/well. Following irradiation, surviving cells were observed, and long-term proliferation was assessed on days 5 and 10 post-exposure. Cell growth was expressed as a percentage relative to non-irradiated controls. The results are presented below.

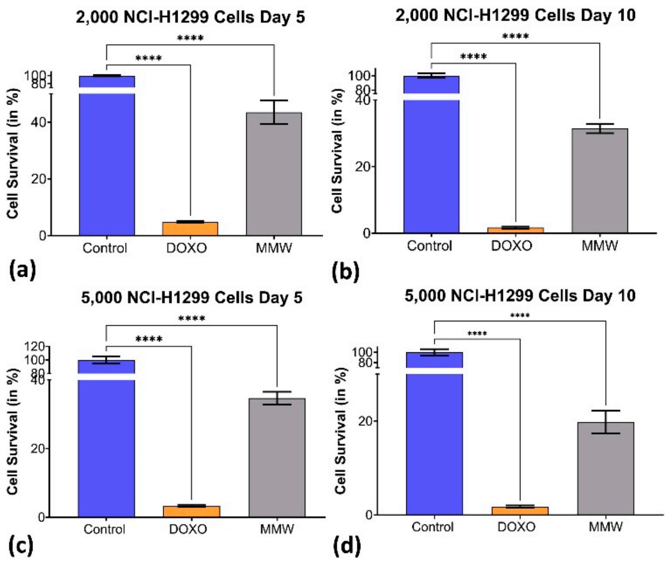

Figure S5. Effect of MMW irradiation on long-term survival of viable NCl-H1299 cells.. NCl-H1299 cells (2.5 × 10^5 cells per condition) were irradiated using a PH antenna without attenuation (90–96 GHz, 4.8 mW, 60 min) and subsequently reseeded at two cell densities, 2.0 × 10^3 and 5.0 × 10^3 cells/well, in a 24-well plate. Long-term survival was assessed on days 5 and 10 post-irradiation and expressed as a percentage relative to non-irradiated controls (set at 100%). **(a)** Survival of 2.0 × 10^3 cells at day 5, **(b)** survival of 2.0 × 10^3 cells at day 10, **(c)** survival of 5.0 × 10^3 cells at day 5, **(d)** survival of 5.0 × 10^3 cells at day 10. The data represented are the mean (± SD) of four independent experiments (n=4). Statistical significance was evaluated by one-way ANOVA at α = 0.5. The significance indicated as: $p < 0.01$ (**), and $p < 0.001$ (***)

As shown in **Figure S5**, MMW irradiation of NCl-H1299 lung cancer cells resulted in a marked reduction in long-term survival compared with non-irradiated controls at both cell densities. For 2.0 × 10^3 cell/well, survival decreased to 43.5 ± 4.08 % on day 5 and 31.5 ± 1.40 % on day 10, compared with approximately 100% survival in control cells ($p < 0.0001$). A similar trend was observed at the

higher cell density of 5.0 × 10^3 cells/well, where survival decreased to 34.7 ± 1.83 % on day 5 and 19.8 ± 2.43 % relative to control ($p < 0.001$). These findings demonstrate that the anti-proliferative effects of MMW irradiation persisted across both cell densities and independently of cell density, indicating a stable and reproducible biological response.

As expected, DOXO treatment produced near-complete loss of surviving cells, consistent with its known strong cytotoxic activity and supporting its use as a positive assay control. However, the primary biological comparisons in this study were performed between MMW-irradiated cells and untreated control cells.

Importantly, this assay complements the SA-β-gal senescence analysis presented in the main manuscript. The observed cell survival assay quantified the fraction of cells capable of continued proliferation, whereas the SA-β-gal assay identified metabolically active senescent cells that survived irradiation but lost proliferative capacity. Together, these assays demonstrated that a substantial fraction of MMW-treated NSCLC cells remained viable yet underwent senescence-associated growth arrest, thereby contributing to the long-term reduction in NSCLC cell expansion.

For subsequent SA-β-gal assays, 1.0 × 10^3 cells/well in 96-well plates were selected to improve assay reproducibility and facilitate fluorometric measurements. The corresponding SA-β-gal activity results for NCl-H1299, A549, and WI38 cells are presented in the main manuscript (**Figure** 7).
